# Effect of *Lacticaseibacillus casei* LC2W Supplementation on Glucose Metabolism and Gut Microbiota in Subjects at High Risk of Metabolic Syndrome: A Randomized, Double-blinded, Placebo-controlled Clinical Trial

**DOI:** 10.1007/s12602-024-10312-5

**Published:** 2024-07-02

**Authors:** Danqi Wang, Xiaohua Wang, Jin Han, Chunping You, Zhenmin Liu, Zhengjun Wu

**Affiliations:** State Key Laboratory of Dairy Biotechnology, Shanghai Engineering Research Center of Dairy Biotechnology, Dairy Research Institute, Bright Dairy & Food Co., Ltd., Shanghai, 200436 PR China

**Keywords:** *Lacticaseibacillus casei* LC2W, Metabolic syndrome, Glucose; Inflammation, Gut microbiome

## Abstract

Metabolic syndrome (MetS) is a global epidemic complex and will cause serious metabolic comorbidities without treatment. A prevention strategy for MetS development has been proposed to modulate gut microbiota by probiotic administration to improve intestinal dysbiosis and benefit the host. *Lacticaseibacillus casei* LC2W has exhibited positive effects in preventing colitis and anti-hypertension in vivo. However, the effect of *L. casei* LC2W on subjects at high risk of MetS is unknown. Here, a randomized, double-blinded, placebo-controlled study was conducted on 60 subjects with high risk of MetS, and the hypoglycemic and hypolipidemic activity and possible pathways of *L. casei* LC2W were inferred from the correlation analysis with gut microbiome composition, function, and clinical phenotypic indicators. The results showed that oral administration of *L. casei* LC2W could exert significant benefits on weight control, glucose and lipid metabolism, inflammatory and oxidative stress parameters, and SCFA production, as well as modulate the composition of gut microbiota. The relative abundance of *Lacticaseibacillus*, *Bifidobacterium*, *Dorea*, and *Blautia* was enriched, and their interaction with other gut microbes was strengthened by oral administration of *L*. *casei* LC2W, which was beneficial in ameliorating gut inflammation, promoting glucose and lipids degradation pathways, thus alleviated MetS. The present study confirmed the prevention effects of *L. casei* LC2W towards MetS from aspects of clinical outcomes and microflora modulation, providing an alternative strategy for people at high risk of MetS.

Trial registration: The study was proactively registered in ClinicalTrial.gov with the registration number of ChiCTR2000031833 on April 09, 2020.

**Supplementary Information:**

The online version contains supplementary material available at 10.1007/s12602-024-10312-5.

## Background

Metabolic syndrome (MetS) is a complex disorder, and its clinical manifestation often comprises abdominal obesity, hyperglycemia, hypertriglyceridemia, lowered high-density lipoprotein cholesterol (HDL-C) levels, and so on [1]. Though the etiology and pathophysiologic mechanisms of MetS are still lack of full understanding and clarity, it is recognized to be a pro-inflammatory and oxidative physiologic state implicated by insulin resistance [2], which confers up soared risk and morbidity of type 2 diabetes mellitus (T2DM) and cardiovascular disease (CVD) [3]. The global prevalence of MetS exhibited a soaring tendency with socioeconomic promotion and living standards improvement. According to the estimation of the International Diabetes Federation (IDF), as much as 25% of the world’s population suffers from MetS [4]. This situation is also serious among Chinese and increases with advanced age. A recent study based on 9258 subjects of the China Health and Retirement Longitudinal Study (CHARLS) datasets indicated the MetS prevalence was about 33.38%, and this figure continuously increased in subjects aged 40–70 years [5]. Liu et al suggested a prevalence of 58.1% in subjects older than 60 years among 2102 subjects [6]. Therefore, intervention with efficacy to alleviate MetS is urgent.

Though ignored in MetS definition, intestinal microbiota might have played a crucial role in the onset and development of MetS according to the last decade’s datasets [7]. Supplement of probiotics is widely accepted as one of the predominant strategies to adjust gut microbiota and to reduce low-grade inflammation states by restoring intestinal microbiota homeostasis and reinforcing the integrity of the intestinal barrier [8]. Several studies have tried to unravel the association of intestinal microbiota with the onset of MetS and explored the potential of probiotics as biotherapeutics for the prevention of MetS. For instance, *Lactiplantibacillus plantarum* was proven to modulate gut microbiota and metabolites in mice [9], accompanied by an alleviated lipid profile as well as glucose metabolism disorders and reduced inflammation markers associated with MetS [10, 11]. It was found that *L. paracasei* NL41 could decrease insulin resistance, oxidative stress status, and LPS-induced inflammation by improving the gut microbiota and preserving intestinal integrity, which prevented T2DM [12, 13]. Moreover, *Bifidobacterium animalis* subsp. *lactis* Bb12 was reported to ameliorate obesity by enriching beneficial bacteria in the gut [14], and effectively decrease triglyceride concentration and homeostasis model of assessment-insulin resistance (HOMA-IR) while significantly increase high-density lipoprotein-cholesterol and quantitative insulin sensitivity check index (QUICKI) [15].

As one of the well-known probiotic strains, *L. casei* LC2W was originally isolated in 2001 and its whole genome sequencing was accomplished in 2011 [16]. *L. casei* LC2W could inhibit *Escherichia coli* O157:H7 colonization and prevent colitis [17], inhibit the inflammatory response and improve acute lung injury, produce exopolysaccharide (EPS), and exhibit anti-hypertensive bioactivity [18]. Besides, *L. casei* LC2W exhibited potential for anti-hyperglycemia, for its strong alpha-glucosidase inhibitory activity in vitro [19]. However, no investigation regarding *L. casei* LC2W on gut microbiome and clinical outcomes has been reported in MetS subjects yet.

In the present study, a randomized, double-blinded, placebo-controlled study was undertaken to investigate the effect of oral administration of *L. casei* LC2W on glucose and lipid profile components as primary endpoints, as well as on inflammation and oxidation stress biomarkers. Moreover, the effects of *L. casei* LC2W on gut microbiome composition, cooperation, and function were also investigated. The hypoglycemic and hypolipidemic activity of *L. casei* LC2W in subjects at high risk of MetS was inferred for its ability to modulate the composition and function of the gut microbiome.

## Methods

### Study Design

A randomized, double-blinded, placebo-controlled study was carried out in 60 subjects with fasting blood glucose higher than 6.1 mmol/L, but lower than 6.9 mmol/L. Enrolled subjects were randomly assigned to *L. casei* LC2W or placebo group. Subjects were required to orally take in 6.0 × 10^11^ colony forming units (CFU) with maltodextrin as the adjuvant or equal weight of maltodextrin for placebo group daily for 6 months. During the study, the dietary intake, sleeping time, physiological exercise, and adverse side reactions of the subjects were recorded weekly with a questionnaire by medical professionals. Subjects forgetting to take in the assigned *L. casei* LC2W powder or placebo for consecutive 3 days and /or ongoing antibiotic treatment during this study were withdrawn. Twenty-eight subjects in the *L. casei* LC2W group and 27 subjects in the placebo group accomplished the study (Table 1).

**Table 1:** Participant disposition

	Group		
	*L. casei* LC2W	Placebo	Total
Enrolled subjects (*n*)	30	30	60
Completed subjects for clinical study (*n*)			
Baseline	30	30	60
3 months	29	28	57
6 months	28	27	55
Completed subjects for microbiome study (*n*)			
Baseline	29	28	57
3 months	26	27	53
6 months	25	26	51

At consumption, subjects took the contents of the sachet directly or with warm water half an hour after the meal. The probiotics powder or placebo were assigned to subjects every 2 months, and the subjects were advised to store the study materials in −20 °C refrigerators throughout the study period.

### Subject Recruitment

Eligible subjects included males and females aged 45–65 years with impaired fasting blood glucose (6.1–6.9 mmol/L) and not under any treatment for blood glucose reduction. Exclusion criteria were diagnosis of diabetes; current treatment for diabetes or gastrointestinal symptoms; presence of active diarrhea; current use of pain relievers such as aspirin or paracetamol; use of laxatives or other supplements to improve digestive gastrointestinal function within 2 weeks before the study entry; history of long-term use of probiotics or prebiotics; ongoing use of antihistamines medication, cough medication, or high-dose vitamin C; use of antibiotics within three months prior to study entry; vaccination for upper respiratory tract infection within 6 months or other vaccination within 15 days prior to study entry; alcohol or drug addiction; and pregnant or breast-feeding women. All recruited subjects were instructed to maintain their usual diet but to avoid consumption of other fermented milk, yogurt, prebiotics, and probiotics products during the study. Subjects withdrew from the study if their glucose kept increasing to the extent that medical treatments were needed.

### Efficacy Evaluation

All efficacy outcomes except fecal biomarkers were measured at baseline, 3 months, and 6 months after probiotic consumption. The primary outcome was fasting blood glucose. Blood samples were drawn between 8 to 10 a.m. following an overnight fast of at least 12 h to quantify glycemic status, lipid concentrations, biomarkers of inflammation, and oxidative stress. The serum insulin was determined by electrochemiluminescence assay (Roche Diagnostics, Germany), and hemoglobin A1c (HbA1c) was measured by high-performance liquid chromatography (Medconn Diagnostics, China). Plasma high-sensitivity C-reactive protein (hs-CRP) concentration was measured by an immunoturbidimetry assay (Goldsite, China). The serum glucose, low-density lipoprotein (LDL), high-density lipoprotein (HDL) cholesterol, total cholesterol (TC), and triglyceride (TG) concentrations were determined by enzymatic kits (Maccura, China); inflammation biomarkers including interleukin-6 (IL-6), interleukin-8 (IL-8), and tumor necrosis factor (TNF-α) were quantified with chemiluminescence methods (Siemens, Germany); oxidative stress biomarkers including superoxide dismutase (SOD) and malondialdehyde (MDA) were determined by spectrophotometric test (Medicom, China).

The oral glucose tolerance test (OGTT) was performed 30 min, 1 h, and 2 h after intake of 75 g of glucose solubilized in 250 mL of warm water. Body weight, height, body mass index (BMI), waist circumference, hip circumference, and waist-to-hip ratio were measured using standard anthropometric methods. Fecal samples were collected at baseline and 3 months post-intervention for short-chain fatty acid measurements with a gas chromatographic method with Agilent 6890N. Subjects were instructed to record their daily food and beverage intake during the 3 days before each visit according to the food models and scales provided. The portion sizes were converted to grams and summarized by food categories. The duration of physical activities in the past week of each visit was also recorded. These data were collected to assess if there was a change in diet and exercise frequency.

### Fecal Microbiome Endpoints

Fecal samples collected at baseline, 3 months, and 6 months were used for DNA extraction according to the instruction of E.Z.N.A.^®^ stool DNA Kit (Omega Biotek, Norcross, GA, USA), and 16S rRNA hypervariable V3–V4 region were amplified by a thermocycler PCR system (GeneAmp 9700, ABI, USA) within 20 µL reaction mixture consisting of 4 µL 5 × FastPfu Buffer, 2 μL 2.5 mM dNTPs, 0.8 μL of each primer (5 μM), 0.4 μL FastPfu polymerase, and 10 ng template DNA. The PCR products were purified by the AxyPrep DNA Gel Extraction Kit (Axygen Biosciences, Union City, CA, USA) and pooled together with equal mole concentrations, followed by Illumina MiSeq platform (Illumina, San Diego, CA, USA) sequencing which generated 2 × 300 paired-end reads according to standard protocols by Sinotech Genome Technology Co., Ltd. (Shanghai, China).

### Statistical Analysis

Demographic and baseline characteristics were summarized to evaluate the significance of the difference between the *L. casei* LC2W and placebo group. For all efficacy outcomes, continuous variables are reported as means ± SD or median (quartiles), and categorical variables are reported as *n* (%). Prior to testing, distributional assumptions for the outcomes were assessed and transformations or nonparametric versions of the tests were used if deemed necessary. The group differences at each visit were evaluated using analysis of variance with adjustment for gender for normal distributed continuous outcomes, Kruskal Wallis test for non-normal data, and chi-square test for categorical data. The within-group between every two visits was evaluated using paired *t*-test for normal distributed continuous outcomes and Wilcoxon signed ranks test for non-normal outcomes. The number and percent of adverse events (AE) and serious adverse events (SAE) were summarized, and the overall AE rate was compared between the two study groups. All efficacy analyses were conducted for subjects who completed the study. The analysis of AE was performed for all subjects. The significance level for statistical tests was set at 0.05. Statistical analyses were performed using the SAS software version 9.4 (SAS Institute Inc., Cary, NC, USA).

Fecal microbiome post-sequencing data were mainly processed with the Mothur software (version v.1.30.1), and the minimum sequencing depth of samples was controlled as 23,984. Usearch (version 7.0) was chosen for operational taxonomic units (OTU) clustering. Bacterial alpha diversity indices Chao 1 were calculated, and Bray-Curtis distance was used to measure community construction according to principal coordinate analysis, comparison between groups at the same visits was performed by Wilcoxon test, while Kruskal Wallis test was within-group between each visit, and the *P* value was adjusted by Bonferroni method. Linear discriminant analysis effect size (LEfSe) [20] was utilized to illustrate the differences in microbiome composition and functional pathways between groups. PICRUST2 [21] was applied to determine the functional attributions by 16S rRNA OTUs, and Metabolic Pathway Database (MetaCyc) pathways were predicted. Random forest (RF) classification algorithm was employed to efficiently identify key species as well as pathways category that were most important for sample classification between groups by R version 4.2.1. Co-occurrence network analysis was adopted to measure the correlations between species which were found in at least five samples and with total relative abundance larger than 0.1%, and visualization was made by Gephi. Spearman correlation was executed for microbes, pathways, and biomarker associations.

### Ethics Statement

The research practices were carried out in accordance with the Code of Ethics of the World Medical Association (Declaration of Helsinki) for experiments involving human beings. The study was reviewed and approved by The Institutional Review Board of the Shanghai Nutrition Society. The study was registered with Clinicaltrials.gov (ChiCTR2000031833), and written informed consent was obtained from all subjects prior to their screening and recruitment in the study. Information about all subjects was kept anonymous, and the privacy rights of subjects were always preserved. All institutional safety standards were adhered to.

### Data Availability Statement

The high-throughput sequencing data amplified in this study have been deposited in the Sequence Read Archive (SRA) database under the BioProject number: PRJNA896346 (https://www.ncbi.nlm.nih.gov/sra/PRJNA896346).

## Results

Of the 60 subjects recruited, mean age was 53.1 years (standard deviation, SD: 4.1 years) and half were men. During the study, three subjects withdrew before the visit at 3 months and another 2 withdrew before the visit at 6 months. The overall dropout rate was 8.3%. All drop-outs were due to personal reasons. Thus, 55 subjects completed the study and were included in the analyses (Table 1). Baseline characteristics were similar between the *L. casei* LC2W and placebo groups (Table S1). During the study, all subjects complied with dietary restrictions and a 72-h dietary recall and 1-week physical activities of each visit was recorded in Tables S2 and S3.

### Anthropometric Measurements

There was no significant difference in anthropometric measurements at baseline (Table S1). However, both body weight (mean change −1.4 kg; 95% CI −2.4, −0.4) and BMI (−0.5 kg/m^2^; −0.8, −0.1) significantly decreased in response to *L. casei* LC2W consumption for 6 months (Table S4), and the weight gain of 6 months *L. casei* LC2W consumption was significantly lower than placebo group (Fig. S1). No significant change was observed in waist circumference, hip circumference, and waist-to-hip ratio (Table S4).

The anthropometric measurements analyses for subgroups of gender exhibited that body weight (female: mean change −1.2 kg; 95% CI −3.3, 1.0; male: mean change −1.5 kg; 95% CI −2.5, −0.5) and BMI (female −0.4 kg/m^2^; −1.2, 0.3; male: −0.5 kg/m^2^; −0.8, 0.1) also significantly decreased in response to *L. casei* LC2W consumption for 6 months. No significant difference was found in anthropometric parameters between female and male subgroups (Table S5).

### Blood and Fecal Biomarkers

**Fig. 1:**
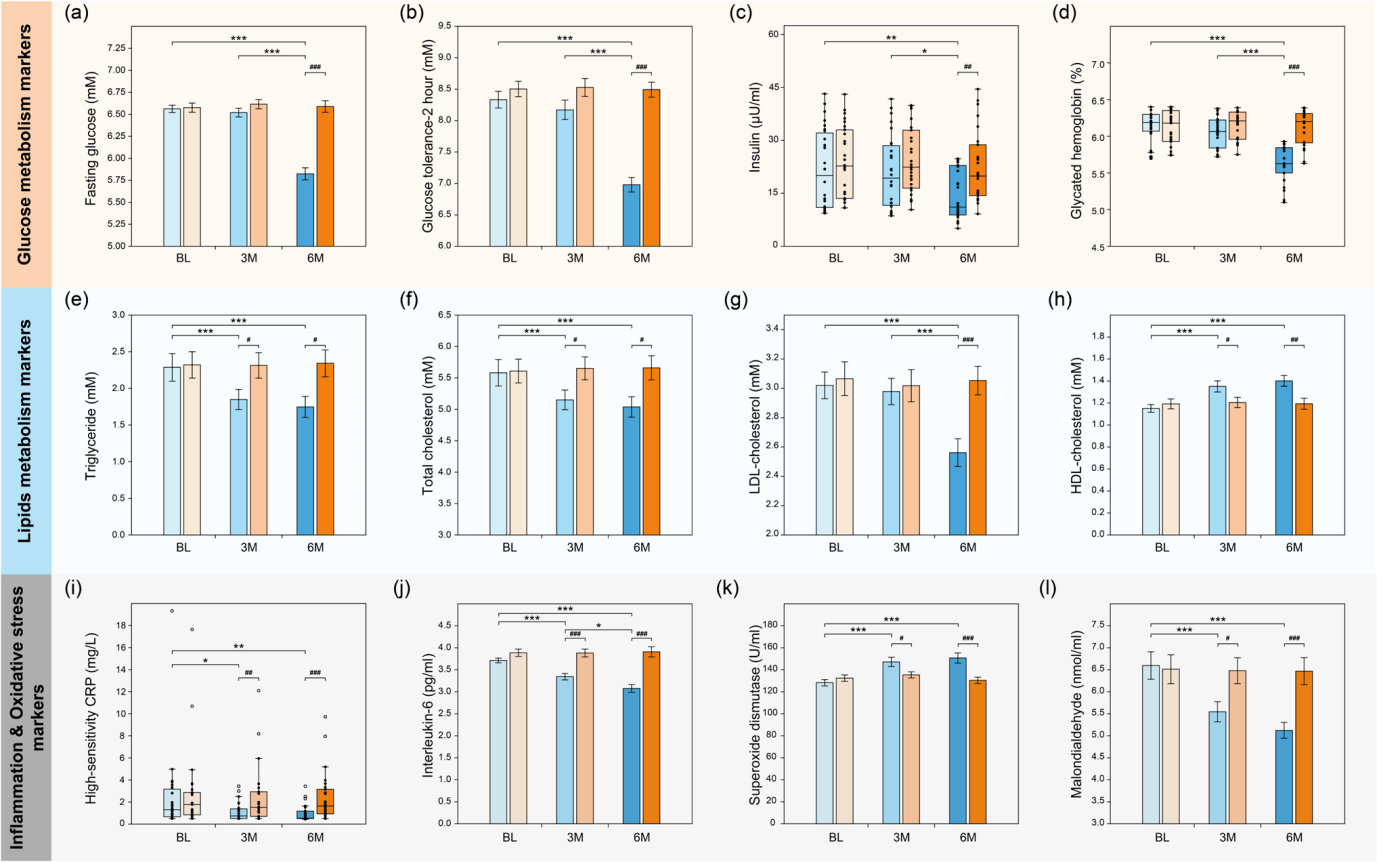
Effects of *L. casei* LC2W supplementation on the change of blood biomarkers. Only biomarkers with significant differences are displayed here. Glucose metabolism markers include fasting glucose (**a**), insulin (**b**), glycated hemoglobin (**c**), and glucose tolerance at 2 h (**d**). Lipid metabolism markers consist of triglyceride (**e**), total cholesterol (**f**), HDL-cholesterol (**g**), and LDL-cholesterol (**h**). Inflammation and oxidative stress biomarkers involve high-sensitivity CRP (**i**), interleukin-6 (**j**), superoxide dismutase (**k**), and malondialdehyde (**l**). Blue color system represents for *L. casei* LC2W group, orange color system stands for placebo group. “*” indicates a significant difference compared to baseline or 3M values, *P < 0.05, **P < 0.01, ***P < 0.001; “#” denotes a significant difference compared to the placebo group, #*P* < 0.05, ##*P* < 0.01, ###*P* < 0.001

There was no significant difference in all blood biomarkers at baseline. After 3 months of *L. casei* LC2W consumption, glucose metabolism markers slightly decreased; however, none of the changes was significant. Significant reductions in serum level of fasting glucose (−0.7 mmol/L; 95% confidence interval, CI −0.9, −0.6), glucose at 30 min (−3.1; −3.8, −2.5), 1 h (−2.7; −3.3, −2.1) and 2 h (−1.4; −1.6, −1.1) in OGTT, insulin (−4.7; −11.6, −2.3), and HbA1c (−0.5%; −0.8, −0.2) were observed after 6 months of *L. casei* LC2W consumption. The levels of all glucose metabolism markers measured were significantly lower than that of the placebo group at 6 months (Fig. 1; Table S6). TG (−0.4 mmol/L; −0.7, −0.2), TC (−0.4 mmol/L; −0.6, −0.3), CRP (−0.3 mg/L; −0.7, −0.1), IL-6 (−0.4 pg/ml; −0.6, −0.2), and MDA (−1.1 nmol/ml; −1.5, −0.6) decreased significantly after 3 months of *L. casei* LC2W intake. Further decreases in these biomarkers were observed at 6 months. The concentrations of these biomarkers of the *L. casei* LC2W group were significantly lower than that of the placebo group at 3 months and 6 months after intervention. At 6 months, the *L. casei* LC2W group also showed significantly lower LDL-cholesterol concentration compared to baseline (−0.5 mmol/L; −0.6, −0.3) and the placebo group. Besides, consumption of *L. casei* LC2W resulted in significant increases in serum HDL-cholesterol (0.2 mmol/L; 0.1, 0.3) and SOD (18.9 nmol/ml; 13.0, 24.7) at 3 months. Similarly, HDL and SOD concentrations of the *L. casei* LC2W group were significantly higher than that of the placebo group at 6 months after intervention. There was no significant change in IL-8 and TFN-α at all visits (Table S7).

As for the subgroups adjust for gender, the majority of the blood biomarker exhibited the consistent results with the adjustment of *L. casei* LC2W to the whole cohorts, without differences between two genders. However, after 6 months *L. casei* LC2W consumption, total cholesterol of female subgroup decreased significantly (−0.92 mmol/L; −1.37, −0.46) and was obvious lower than male subgroup (−0.35; −0.51, −0.16), while the level of IL-6 in male subgroup (−0.82 pg/ml; −1.15, −0.49) was significantly lower than that in female subgroup (−0.30; −0.44; −0.16) (Table S7), showing a slightly difference response in subjects intaking of *L. casei* LC2W.

**Fig. 2:**
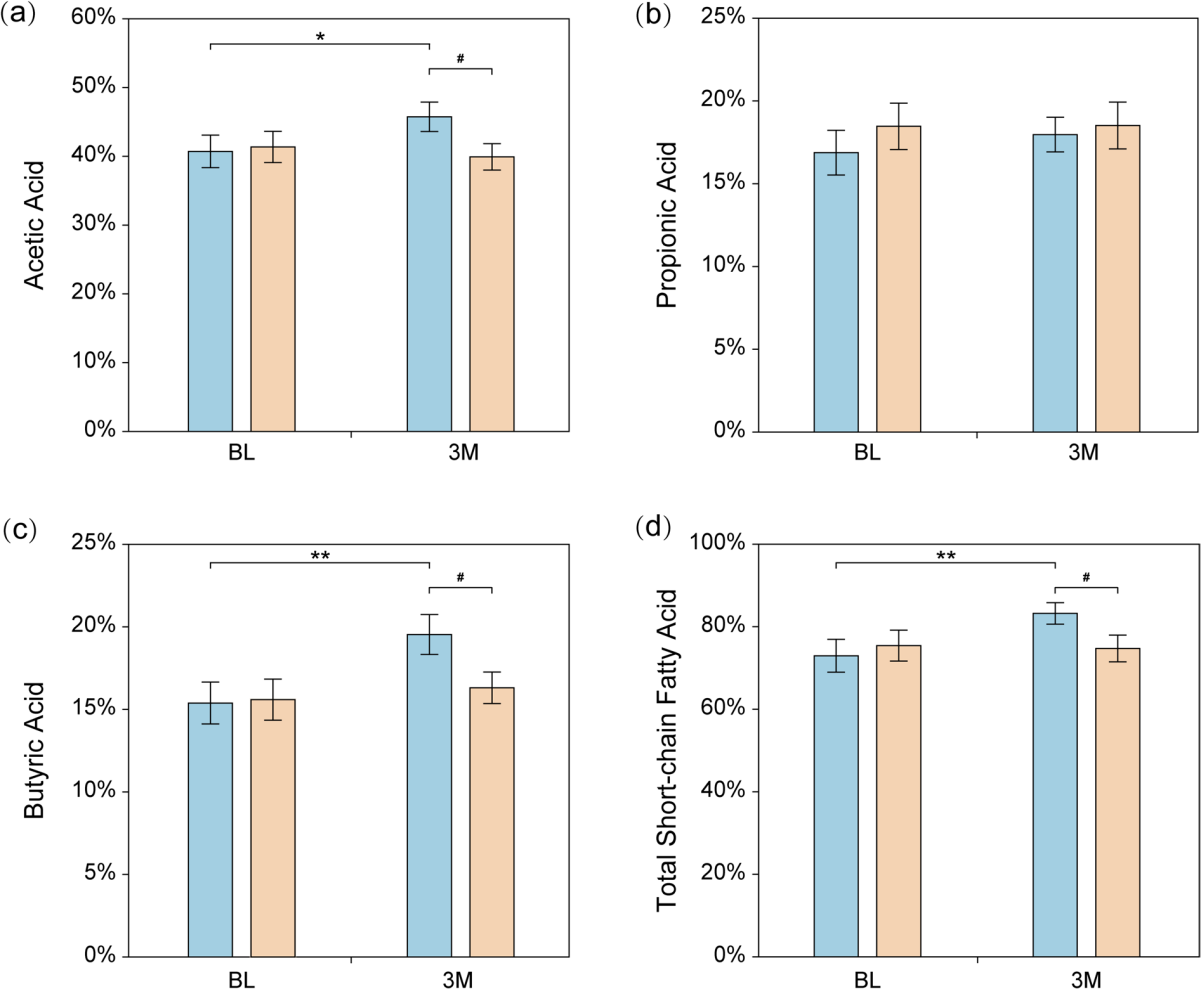
Effects of *L. casei* LC2W supplementation on the change of fecal biomarkers. Acetic acid (**a**), propionic acid (**b**), butyric acid (**c**), and total short-chain fatty acid (**d**). Blue color system represents for *L. casei* LC2W group; orange color system stands for placebo group. “*” indicates significant different compared to baseline values, *P < 0.05, ** *P*< 0.01. “#” denotes a significant difference compared to placebo, #*P* < 0.05

The fecal concentration of acetic acid (5.0%; 95% CI 1.2, 8.8), butyric acid (4.2%; 2.1, 7.3), and total SCFA (10.3%; 3.4, 16.8) increased significantly after 3 months of *L. casei* LC2W consumption, and the post-intervention values were significantly higher than that of the placebo group (Fig. 2). There were no significant changes in propionic acid in both study groups.

### Microbiome Composition and Correlation Profiling

**Fig. 3:**
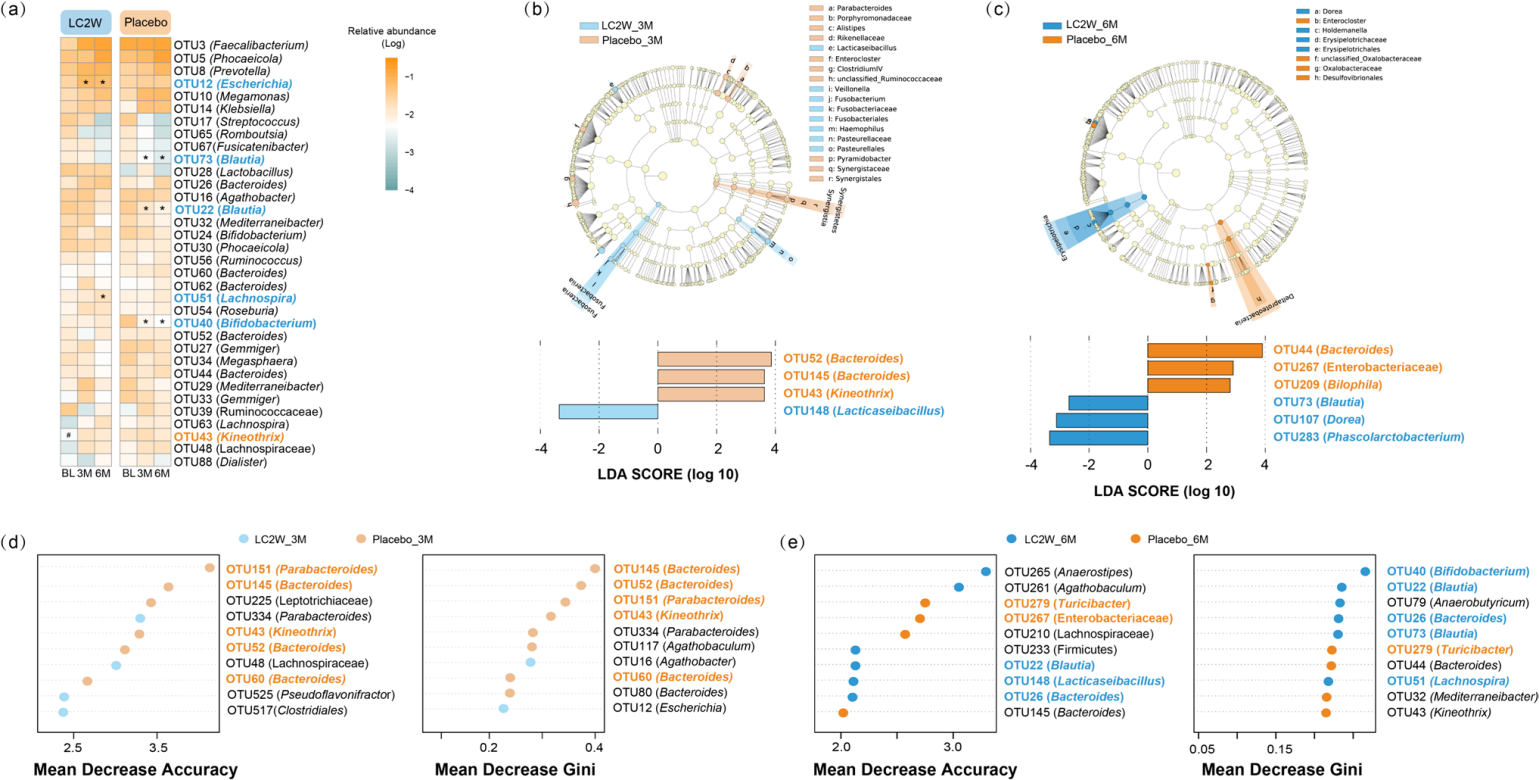
Bacterial heatmap of major 34 OTUs’ relative abundance according to six groups based on *L. casei* LC2W applied or not and sampling time (**a**). “*” indicates *P* < 0.05 versus baseline in the Kruskal-Wallis test adjusted by the Bonferroni method. “#” denotes *P* < 0.05 versus placebo group by Wilcoxon test. Distinct bacterial features were identified by LEfSe analysis between *L. casei* LC2W and placebo datasets in 3 months (**b**) and 6 months (**c**). The cladogram showed the taxonomical levels from phylum down to genus and the histogram revealed OTU levels. Top 10 OTUs with the highest mean decrease accuracy and Gini scores by Random Forest classification model for 3 months (**d**) and 6 months (**e**) datasets. The color of the dots represents the enrichment of OTUs in each group by the median

Regarding the bacterial diversity in the fecal samples, there was no significant difference in both alpha and beta diversity between the two groups at different sampling times. For fecal microbiome composition, no significant differences were found between the two groups sampled at both 3 months and 6 months at the phylum level. However, for the overall datasets, LEfSe identified that genus *Lacticasebacillus*, *Veillonella*, *Fusobacterium*, and *Haemophilus* were enriched after 3-month intervention with *L. casei* LC2W, while the relative abundance of *Parabacterioldes*, *Alistipes*, *Pyramidobacter*, *ClostridiumIV*, and unclassified_Ruminococcaceae was significantly higher in the placebo 3 months datasets (LDA score > 2, *P* < 0.05; Fig. 3b). After 6 months of intervention with *L. casei* LC2W, the relative abundance of *Dorea* was significantly improved, while the Desulfovibrionaceae family showed predominance in the placebo 6 months cohort (LDA score > 2, *P* < 0.05; Fig. 3c). Further OTU level comparisons were executed on 34 major OTUs with relative abundance > 0.5% for microbiome composition analysis. According to the heatmap, there were no significant differences in major OTUs except for *Kineothrix* OTU43 in both groups at baseline (Fig. 3a). However, it was exhibited that the relative abundance of *Bifidobacterium* OTU40 and 2 *Blautia* OTUs (OTU22 and OTU73) was obviously diminished in placebo datasets along sampling time (Kruskal-Wallis test, *P* < 0.05). In contrast, their relative abundance showed no significant differences after *L. casei* LC2W intervention, and the relative abundance of *Bifidobacterium* OTU40 was even elevated at 6 months versus baseline (Fig. 3a). Besides, after 6 months of *L. casei* LC2W intake, the relative abundance of *Lachnospira* OTU51 was also significantly increased, while there was no obvious increment in the placebo dataset. For the overall datasets, a *Lacticaseibacillus* OTU148 was promoted by 3 months of *L. casei* LC2W intake, while two *Bacteroides* OTUs (OTU52 and OTU145) as well as *Kineothrix* OTU43 were increased in 3 months placebo groups (Fig. 3b). For 6 months dataset, *Blautia* OTU73, *Dorea* OTU107, and *Phascolarctobacterium* OTU283 were elevated by intaking *L. casei* LC2W, while *Bacteroides* OTU44*,* Enterobacteriaceae OTU267, and *Bilophila* OTU209 possessed a high abundance in placebo groups (Fig. 3c). The random forest classification was established, and top 10 OTUs with the highest mean decrease accuracy and mean decrease gini value were exhibited; alike the results obtained by LEfSe, an increase in the relative abundance of *Escherichia* OTU12 and *Agathobacter* OTU16, as well as *Blautia* OTU22, *Bifidobacterium* OTU40, *Bacteroides* OTU26, and *Anaerostipes* OTU265, was selected by the RF model in the feces after 3 months and 6 months of *L. casei* LC2W intake, respectively. The enrichment of *Parabacteroides* OTU151, *Bacteroides* OTU60, and *Turicibacter* OTU279 was identified in 3 months as well as 6 months placebo datasets (Fig. 3 d and e).

According to the results of microbiome composition analysis and random forest classification, *Blautia* and *Bifidobacterium* OTUs were enriched by *L. casei* LC2W consumption for both female and male subgroups, while *Parabacteroides* and *Turicibacter* OTUs possessed higher relative abundance in female and male subjects in the placebo group, respectively (Fig. S2).

**Fig. 4:**
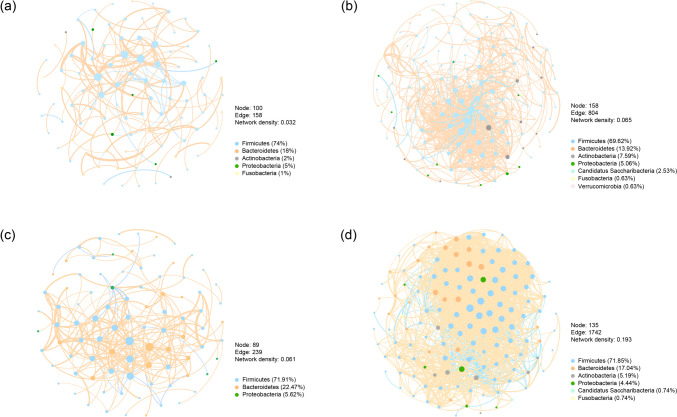
Co-occurrence networks established by OTUs appeared in at least five samples with a total relative abundance larger than 0.1% for the baseline placebo group (**a**), baseline *L. casei* LC2W group (**b**), 6 months placebo group (**c**), and 6 months *L. casei* LC2W group (**d**). The nodes represent 16S rRNA OTUs with the size reflecting the average weighted degree in the co-occurrence pattern. The edges represent the correlation between nodes, and the thickness stands for Spearman correlation coefficients; the edges’ color indicates the correlation was positive (orange) or negative (blue)

To further explore the microbiome correlation and connection, concurrence networks were established for both the *L. casei* LC2W and placebo groups at baseline and 6 months (Spearman |*r*| > 0.5, *P* < 0.05; Fig. 4). Although the number of nodes in the network constructed by *L. casei* LC2W datasets is less than those of placebo datasets at both baseline and 6 months, intervention of *L. casei* LC2W promoted the network complexity as the number of edges increased by 2.16-fold, whereas the edge numbers of placebo group only increased by 1.51-fold. Moreover, the network density was also increased after intake of *L. casei* LC2W compared to the placebo group, indicating a relatively strong regulating effects on microbiome community after *L. casei* LC2W intake. Furthermore, the OTUs significantly enriched by *L. casei* LC2W intake also showed up in the *L. casei* LC2W co-occurrence pattern and absent in placebo network, with the average weighted degree increased by 2.04-fold, 3.84-fold, 5.25-fold, 6.46-fold, and 14.13-fold for *Blautia* OTU22, *Lachnospira* OTU51, *Bifidobacterium* OTU40, *Bacteroides* OTU26, and *Dorea* OTU107, respectively. Notably, *Anaerostipes* OTU265, which disappeared in *L. casei* LC2W baseline co-occurrence network, possess a high average weighted degree of 25.24 in the network of fecal microbiota with *L. casei* LC2W intake for 6 months, suggesting *L. casei* LC2W intake could not only stimulate the specific beneficial species such as *Blautia* OTU22, *Lachnospira* OTU51, *Bifidobacterium* OTU40, *Bacteroides* OTU26, and *Dorea* OTU107 but also enhance their interfere towards microbiome interaction, especially for *Anaerostipes* OTU265.

### Microbiome Function Prediction

**Fig. 5:**
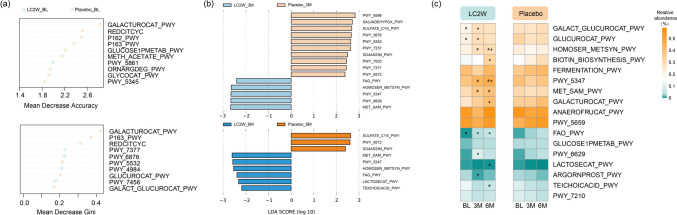
Top 10 pathways with the highest variable importance selected by random forest classification model in baseline (**a**). The color of the dots represents the predicted pathways that were predominant in *L. casei* LC2W (blue) and placebo (orange), respectively. LEfSe histogram depicting discriminatory pathways between the *L. casei* LC2W (blue) and placebo (orange) cohorts in 3 months and 6 months (**b**). The median proportions in pathways differences between the two datasets at different times (**c**). “*” indicates *P* < 0.05 versus baseline in the Kruskal-Wallis test adjusted by the Bonferroni method. “#” denotes *P* < 0.05 versus placebo group by Wilcoxon test

According to the MetaCyc pathway abundance table generated by PICRUST2 for OTUs in each dataset, a total of 4 pathways were significantly enriched in the *L. casei* LC2W group after both 3 months and 6 months of intaking (LDA score > 2 and *p* < 0.05), including L-methionine and S-adenosyl-L-methionine biosynthesis (MET-SAM-PWY, PWY-5347, and HOMOSER-METSYN-PWY) and fatty acid degradation (FAO-PWY) (Fig. 5b). Besides, L-tryptophan biosynthesis (PWY-6629) was significantly elevated by *L. casei* LC2W intake for 3 months, while lactose and galactose degradation (LACTOSESCAT-PWY), as well as teichoic acid biosynthesis (TEICHOICACID-PWY), exhibited promotion after 6 months consumption (Fig. 5b). Of note, glucose and glucose-1-phosphate degradation (GLUCOSE1PMETAB-PWY), which occurred as the major characteristic pathway in the placebo group at baseline (Fig. 5a), was increased by *L. casei* LC2W intake, and its relative abundance in *L. casei* LC2W group has exceeded placebo groups at 3 months and 6 months. Although three sugar acid degradation pathways including GLUCUROCAT-PWY, GLUCUROCAT-PWY, and GALACTUROCAT-PWY were overabundant in the baseline placebo group (LDA score > 2 and *P* < 0.05), their predominance in placebo group were disappeared, as 3 months of *L. casei* LC2W intake enhanced the first two pathways, while 6 months of *L. casei* LC2W intake promoted the last pathway significantly, even surpass the placebo group (Kruskal-Wallis test, *P* < 0.05; Fig. 5c). As for homolactic fermentation (ANAEROFRUCAT-PWY) and biotin biosynthesis (BIOTIN-BIOSYNTHESIS-PWY) which were prevailing in the placebo group at baseline, 6 months intake of *L. casei* LC2W has significantly enriched these two pathways and surpassed the placebo group (Kruskal-Wallis,* P* < 0.05; Fig. 5c). When it comes to the placebo group, three pathways involving sulfur compound metabolism (SULFATE_CYS_PWY and SO4ASSIM_PWY) and glycosaminoglycan degradation (PWY-6572) possess greater proportions in both 3 and 6 months.

### Association of Bacterial OTUs with Biomarkers and Function

**Fig. 6:**
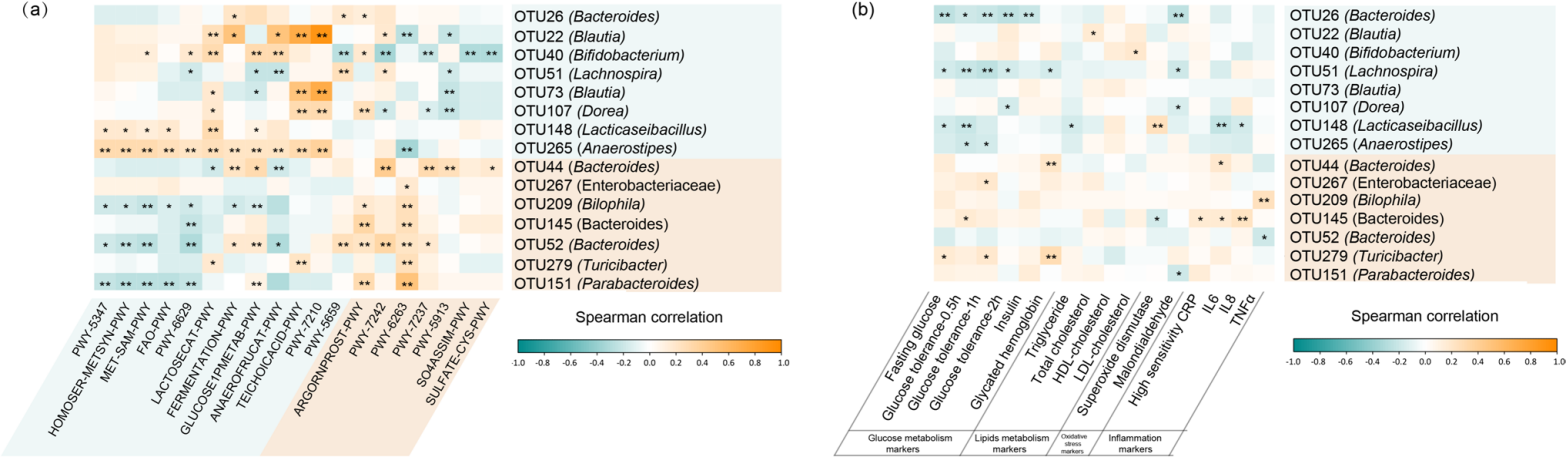
Association between major discriminatory microbe OTUs and specific pathways of differences (**a**). The relationship of 15 OTUs and 19 MetaCyc pathways in the *L. casei* LC2W and placebo cohorts was reflected by heatmap with orange and cyan indicating positive and negative Spearman correlation coefficients, respectively. Orange and blue shades represent the microbes, and the pathways were enriched in the placebo and *L. casei* LC2W groups, respectively. “**” and “*” denote* P* < 0.01 and *P* < 0.05, respectively. Association between major discriminatory microbe OTUs and 16 biomarkers (**b**). The relationship of 15 OTUs and 16 biomarkers in the *L. casei* LC2W and placebo cohorts were reflected by heatmap with orange and cyan indicating positive and negative Spearman correlation coefficients, respectively. Orange and blue shades represent the microbes that were enriched in the placebo and *L. casei* LC2W groups, respectively. “**” and “*” denote* P* < 0.01 and *P* < 0.05, respectively

Spearman correlation analysis was carried out to figure out the exact correlation between the bacterial OTUs, pathways, and biomarkers that differed in *L. casei* LC2W and placebo groups (Fig. 6). The relative abundance of *Anaerostipes* OTU265, *Bifidobacterium* OTU40, and *Lacticaseibacillus* OTU148 which were enriched by *L. casei* LC2W intake was positively correlated with L-methionine and S-adenosyl-L-methionine biosynthesis pathways, while the *Parabacteroide* OTU151, *Bacteroides* OTU52, and *Bilophila* OTU209 enriched in the placebo group exhibited opposite trend (Fig. 6a). As for fatty acid degradation, the majority of OTUs with higher relative abundance in the placebo dataset possessed a negative correlation trend, while *Anaerostipes* OTU265 and *Lacticaseibacillus* OTU148, which were enriched in *L. casei* LC2W group, showed promoting effects. A total of 6 OTUs predominant in the *L. casei* LC2W group actively correlated with lactose and galactose degradation; however, there were only one OTU enriched in the placebo group showing a positive correlation and five of the other placebo-enriched OTUs exhibiting a negative tendency with this pathway. Three OTUs with higher relative abundance after *L. casei* LC2W intake including *Blautia* OTU22, *Anaerostipes* OTU265, and *Bifidobacterium* OTU40 were actively related to fermentation and glucose degradation pathways. However, no positive relationship was found between fermentation pathway and OTUs with higher relative abundance in the placebo group. A total of four OTUs enriched in the *L. casei* LC2W group positively correlated with an increased abundance of teichoic acid and pyrimidine deoxyribonucleotide biosynthesis (*P* < 0.001). On the contrary, OTUs promoted in placebo datasets do not show a close connection between these two pathways except for *Turicibacter* OTU279. As for the pathways with high levels in the placebo group, microbes enriched in *L. casei* LC2W group exhibited a weaker connection or opposite negative trend compared to OTUs promoted in the placebo group.

Bacterial OTUs promoted by *L. casei* LC2W intake including *Bacteroide* OTU26, *Lachnospira* OTU51, *Lacticaseibacillu* OTU148, *Dorea* OTU107, and *Anaerostipes* OTU265 exhibited negative correlations with one or more glucose metabolism biomarkers. However, bacterial OTUs with higher abundance in placebo groups such as *Bacteroides* OTUs (OTU145 and OTU44), and *Turicibacter* OTU279 exhibited positive trends with these biomarkers (Fig. 6b). As for lipids metabolism markers, *L. casei* LC2W-enriched OTUs were negatively correlated with triglyceride and positively correlated with HDL-cholesterol, with *Lacticaseibacillus* OTU148 displaying negative effects with triglyceride, total, and LDL-cholesterol, while most of the placebo-enriched OTUs exhibited the opposite trends although not all of the correlation showed significances. A similar situation was also found in biomarkers involving inflammation, as most of the bacterial OTUs promoted by *L. casei* LC2W intake were negatively correlated with pro-inflammatory factors while bacterial OTUs abundant in the placebo group showed positive correlation. When it comes to malondialdehyde, a biomarker related to oxidative stress, three OTUs including *Bacteroide* OTU26, *Lachnospira* OTU51*,* and *Dorea* OTU107 exhibited a pronounced negative correlation in the *L. casei* LC2W group, while only *Parabacteroide* OTU151 revealed a negative relationship in the placebo group.

### Adverse Events

There were 28 adverse events during the study (Table S8). However, no significant difference was found between the two study groups in the overall AE rate (*P* = 0.438). None of the adverse events was judged to correlate to the tested the probiotic product by medical professionals. No serious AE was recorded during the study.

## Discussion

Metabolic syndrome (MetS), which manifests as central obesity together with high levels in triacylglycerols, blood pressure, fasting plasma glucose, and low level in high-density lipoprotein cholesterol, leads to an increasing risk of both type 2 diabetes mellitus and cardiovascular disease. In the current study, subjects aged 45–65 years with high risks of MetS were administered lyophilized *L. casei* LC2W powder with maltodextrin as an adjuvant for 6 months, to explore its amelioration effects on MetS from both clinical and microbiome indicators.

As for the clinical outcomes, intake of *L. casei* LC2W could decrease the body weights as well as BMI index (Table S4), and reduce all six glucose metabolism markers measured and lipid markers while elevating HDL-cholesterol of subjects of high risk of MetS (Fig. 1; Table S6). Besides, *L. casei* LC2W significantly reduced the inflammation markers including hsCRP and IL-6. IL-6 was reported to induce insulin resistance by inhibiting transcription or reducing phosphorylation of insulin receptor substrate-1 [22], while elevated hsCRP was a prestigious biomarker in inflammation and associated tightly with cardiovascular risk [23]. Hence, *L. casei* LC2W could benefits subjects with high risks of MetS via reducing inflammation intensity. Meanwhile, intake of *L. casei* LC2W also decreased the serum MDA level while increasing serum SOD level, significantly. MDA is the end product of lipid peroxidation and hallmark of elevated oxidative stress, while SOD could help eliminate harmful peroxides generated during metabolic processes [24, 25]. Their changes indicated that intake of *L. casei* LC2W could effectively reduce oxidative stress and hence improve glucose and lipid metabolism in the body. Moreover, intake of *L. casei* LC2W also significantly increased the fecal content of SCFAs including acetic acid and butyric acid (Fig. 2), which exhibited significant negative correlation with some of the metabolism and inflammation markers including 2-h-glucose tolerance, LDL-cholesterol, and hsCRP (Table S9), and positively correlated with *Blautia* OTU73 and *Lactobacillus* OTU28 (Table S10) and fermentation pathway (Table S11). SCFAs could serve as signaling molecules between the gut microbiome and the host [26] and play a key role in the homeostasis of glucose metabolism by reducing the oxidative stress of β-cells in the pancreatic islet, increasing insulin release, and reducing the expression of pro-inflammatory cytokines and anti-lipolysis [27].

Besides clinical biomarkers, we also analyze the microbiome composition, interaction, and function to infer the ability of glucose and lipid modulation of *L. casei* LC2W from a microbiome perspective. Results from previous reports suggested that different OTUs within the same genus might play discriminate roles with some providing protective effects while others exerting detrimental impacts to the host health [28]. Thus, in our study, the priority on analysis of microbiota composition was focused on OTU level, and we found that *L. casei* LC2W application not only increased the relative abundance of specific OTUs but also enhanced their interference towards microbiome interaction (Fig. 4). It is shown that the *Lacticaseibacillus* OTU148 was enriched after *L. casei* LC2W intervention, and *L. casei* LC2W intake could also enhance the relative abundance of *Bifidobacterium* OTU40, members of which were reported to effectively lower cholesterol and display anti-inflammatory activity in high-fat diet feed mouse model [29]. Besides, *L. casei* LC2W intake promoted the relative abundance of *Anaerostipe* OTU265, which was reported to protect animals from food anaphylaxis in inbred germ-free mice and thus be considered a candidate of the next generation probiotics [30]. According to the correlation analysis, the above three OTUs exhibited negative trends towards IL-6 (with *Lacticaseibacillus* OTU148 exhibited significances; Fig. 6b) and were positively correlated with L-methionine and S-adenosyl-L-methionine biosynthesis pathways (Fig. 6a). L-methionine was reported to improve intestinal barrier integrity [31], while S-adenosyl-L-methionine could play diversified roles in health maintenance, including enhanced energy production in cells as well as insulin sensitivity [32–34]. Thus, the underlying mechanism for *L. casei* LC2W to ameliorate the risk of MetS might be correlate with its anti-inflammatory activity. In addition, *Anaerostipes* OTU265 and *Lacticaseibacillus* OTU148 possess a positive correlation trend with fatty acid degradation (Fig. 6a), and *Lacticaseibacillus* OTU148 display negative effects with triglyceride (*P* < 0.05; Fig. 6b), total, and LDL-cholesterol, suggesting a possible pathway of *L. casei* LC2W in decreasing serum free fatty acid and triglycerides.

Previous studies claimed that depletion of *Blautia* species (especially *Blautia luti* and *Blautia wexlerae*) was positively correlated with insulin resistance in obese individuals [35], and oral administration of *Blautia* to mice could eliminate the symptoms of both obesity and diabetes [36]. Due to their antibacterial activity, anti-inflammatory effects, and inverse correlation with aging, members of *Blautia* were proposed as the next generation of probiotics [37, 38]. Members in the *Dorea* genus, most of which exhibited anti-inflammatory activity in vivo, were often found tightly associated with *Blautia* as the gas produced by *Dorea* from carbohydrates could be further utilized by *Blautia* [39]. A similar situation was also observed in subjects with *L. casei* LC2W intake in this study, as the abundance of two *Blautia* OTUs and *Dorea* were promoted, simultaneously (Fig. 2a and c). According to the correlation analysis, three OTUs with higher relative abundance after *L. casei* LC2W intake including *Blautia* OTU22, *Anaerostipes* OTU265, and *Bifidobacterium* OTU40 were actively related to acids fermentation and glucose degradation, and a total of 6 OTUs predominant in *L. casei* LC2W group actively correlated with lactose and galactose degradation (Fig. 6a). Moreover, *Lacticaseibacillus* OTU148, *Dorea* OTU107, and *Anaerostipes* OTU265 exhibited negative correlations with one or more glucose metabolic biomarkers (Fig. 6b). Together, it is indicated that *L. casei* LC2W intake boosted pathways of homolactic fermentation, glucose, and lactose degradation, which helps to decrease the serum glucose. *Lacticaseibacillus casei* LC2W consumption also enhanced mixed acid fermentation and its principal products short-chain fatty acids, which was proved to be negatively correlated with obesity degree [40].

Interestingly, *L. casei* LC2W seemed to exert different effects within *Bacteroidetes* OTUs, with the relative abundance of *Bacteroides* OTU26 promoted, while three other *Bacteroidetes* OTUs (OTU44, OTU52, and OTU145) decreased (Fig. 2). Moreover, *Bacteroides* OTU26 was negatively correlated to glucose metabolism and MDA, while the three decreased *Bacteroidetes* OTUs exhibited a contrasting trend, and some of them were positively correlated with glucose as well as inflammatory biomarkers (Fig. 6b). Therefore, one of the underlying pathways for the hypoglycemic effect of *L. casei* LC2W might stem from the modulation of the *Bacteroides* in the gut microbiome composition. Controversial roles regarding *Bacteroides* species to their host health have been reported in the last decade. Li et al found that *Bacteroides* were associated with T2DM risk in obese individuals by promoting IL-17-producing cell expansion in the peripheral blood [41], and were prevalent in patients with colitis [42, 43]. However, *Bacteroides stercoris* was infrequently isolated in clinical samples and with less apparent association with diseases [44], and showed a positive correlation with lower diastolic blood pressure [45]. As *Bacteroides* OTU26 was most closely related to *Bacteroides stercoris*, its effects in MetS alleviation need to be further verified.

In contrast to the placebo group, intake of *L. casei* LC2W reduced the relative abundance of *Bilophila* OTU209 belonging to the family Desulfovibrionaceae; *Bilophila* OTU209 exhibited a negative correlation with L-methionine and S-adenosyl-L-methionine biosynthesis pathways, and showed significant positive correlation with TNFα (Fig. 6). A higher relative abundance of family Desulfovibrionaceae has been reported in obese humans [46] and mice [47], which was also identified as an important endotoxin producer in constipation patients and could aggravate the symptoms in these patients by reducing intestinal hormone secretion and destroying intestinal integrity [48]. Furthermore, administration of *L. casei* LC2W also reduced the relative abundance of *Turicibacter* OTU279, which exhibited a tight correlation with glucose and HbA1c concentrations (Fig. 6b). *Turicibacter* has been reported to express an analog protein to serotonin transporter with strong pro-inflammatory activity [49, 50], which might contribute to the development of MetS.

**Fig. 7:**
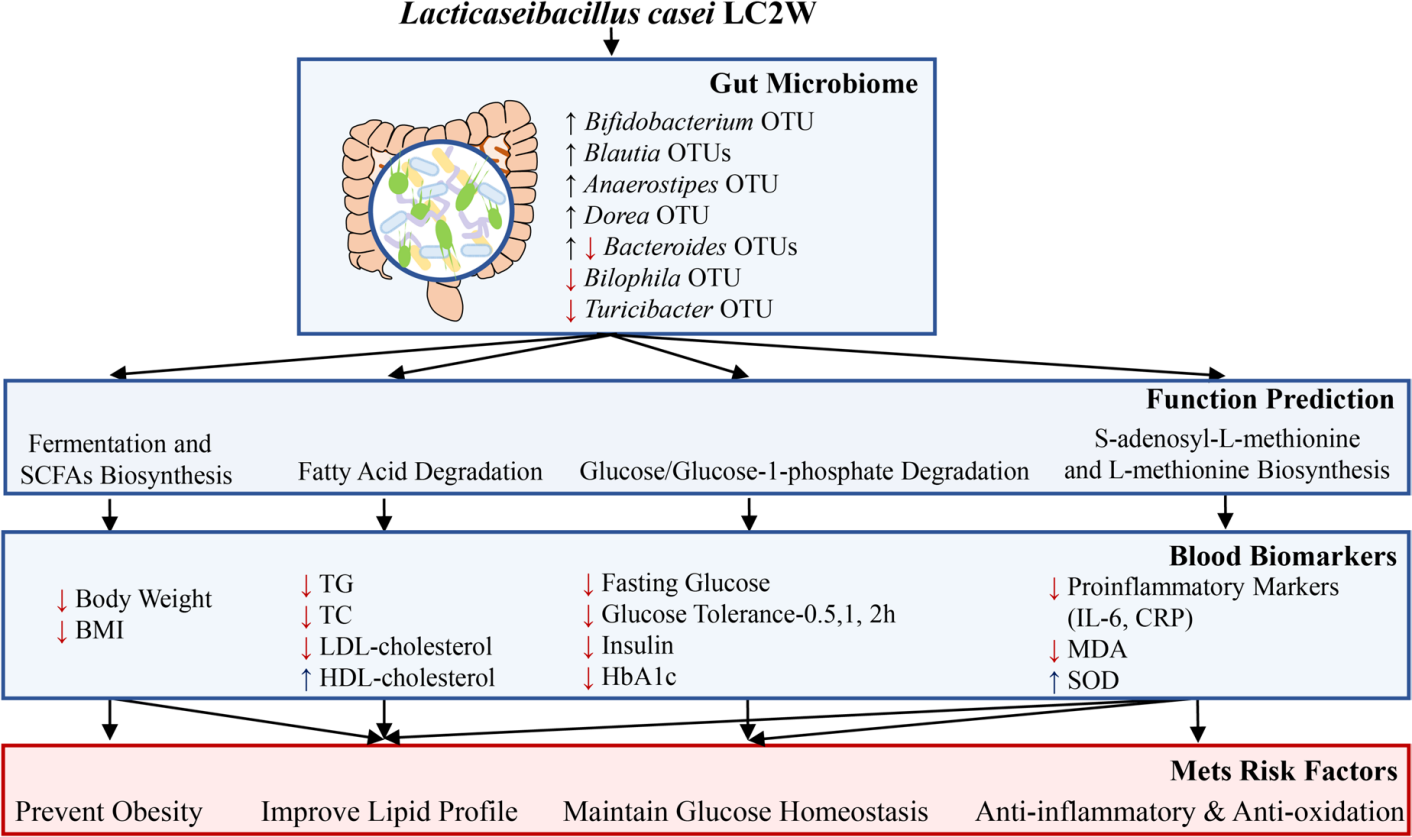
Schematic representation of the impact on gut microbiome modulation and glucose as well as lipids metabolism among subjects at high risk of metabolic syndrome after *L. casei* LC2W supplementation

Overall, this study is the first clinical study to investigate the effects of *L. casei* LC2W on both the clinical indicators including glucose and lipids metabolism, and the gut microbiome profile among subjects at high risk of MetS. Although previous studies have reported that probiotics or synbiotics administration could alleviate the MetS, the subjects’ number, and phenotypic indicators were limited compared to this study [51]. Besides, although some studies involve the variation of both clinical and microbiome indicators at the same time, the OTU level analysis, functional analysis, and correlation analysis between indicators were ignored [52, 53]. Our study claimed that intake of *L. casei* LC2W could significantly decrease body weight and BMI, reduce glucose and lipid levels in serum, and eliminate the pro-inflammatory cytokines and oxidative stress compared to the placebo group. The amelioration of MetS by *L. casei* LC2W might stem from its ability to enhance the relative abundance of beneficial bacteria and their interference towards microbiome interaction, boosting the predicted glucose and lipid degradation pathways in the gut microbiota (Fig. 7). Our study has several strengths; for instance, most clinical studies pay attention to the microbiota shifts in genus level, ignoring the functional discrepancy of OTUs within the same genus, while we focus more on OTU level analysis except for genus level. Besides, in addition to the change in the relative abundance of different bacterial taxa, the interaction among the OTUs was also verified.

### Limitations and Innovations

In the present study, the effects of probiotics on subjects with high MetS risk over 6 months employing serum biomarkers, intestinal microbiome, and their predicted function as the major endpoints were intensively investigated. The correlation between different endpoints has also been dig out, which is helpful for the follow-up mechanism research. However, the analysis of SCFAs was only conducted at baseline and 3 months. Besides, the function of bacterial taxa enriched or diminished by *L. casei* LC2W administration needs to be verified.

## Conclusion

As a conclusion, in the randomized, double-blinded, placebo-controlled study, intake of *L. casei* LC2W for 6 months could significantly improve the symptoms in subjects with a high risk of MetS, including fasting blood glucose, serum lipid level, and BMI. Intake of *L. casei* LC2W could also ameliorate the inflammatory intensity and oxidative stress, and elevate SCFAs production in the feces. The gut microbiota shifted to a composition with a relatively high abundance of bacterial OTUs belonging to *Lacticaseibacillus*, *Bifidobacterium*, *Dorea*, *Blautia*, and their interaction with other gut microbes was boosted as well, which was beneficial in improving chronic inflammation, decreasing serum glucose and lipids, thus alleviating MetS, while the relative abundance of bacterial OTUs belongs to *Bilophila*, *Turicibacter*, etc., was decreased.

## Supplementary Information

Below is the link to the electronic supplementary material.

Supplementary file1 (DOCX 429 KB)

## Data Availability

The authors declare that the data supporting the findings of this study are available within the article.

## Abbreviations

*L. casei*: lacticaseibacillus casei
MetS: Metabolic syndrome
SCFAs: Short-chain fatty acids
HDL-C: High-density lipoprotein cholesterol
T2DM: Type 2 diabetes mellitus
CVD: Cardiovascular disease
CHARLS: China Health and Retirement Longitudinal Study
LPS: Lipopolysaccharide
HOMA-IR: Homeostasis model of assessment-insulin resistance
QUICKI: Quantitative insulin sensitivity check index
EPS: Exopolysaccharide
CFU: Colony forming units
FBG: Fasting blood glucose
HbA1c: Hemoglobin A1c
hs-CRP: High-sensitivity C-reactive protein
LDL-C: Low-density lipoprotein cholesterol
TC: Total cholesterol
TG: Triglyceride
IL-6: Interleukin-6
IL-8: Interleukin-8
TNF-α: Tumor necrosis factor
SOD: Superoxide dismutase
MDA: Malondialdehyde
OGTT: Oral glucose tolerance test; BMI: body mass index
AE: Adverse events
SAE: Serious adverse events
OTU: Operational taxonomic units
LEfSe: Linear discriminant analysis effect size
RF: Random forest
PICRUSt2: Phylogenetic Investigation of Communities by Reconstruction of Unobserved States 2
