# Supplementary material for: Effect of Lacticaseibacillus casei LC2W Supplementation on Glucose Metabolism and Gut Microbiota in Subjects at High Risk of Metabolic Syndrome: A Randomized, Double-blinded, Placebo-controlled Clinical Trial
Source: Probiotics Antimicrob Proteins. 2024 Jul 2;17(6):3811–25. doi: 10.1007/s12602-024-10312-5 (PMC12634783; doi:10.1007/s12602-024-10312-5)
Supplement: Supplementary file 1 — Supplementary file1 (DOCX 429 KB) [file 12602_2024_10312_MOESM1_ESM.docx]

**Supplementary Files**

**Effect of *Lacticaseibacillus casei* LC2W Supplementation on Glucose Metabolism and Gut Microbiota in Subjects at High Risk of Metabolic Syndrome: A Randomized, Double-blinded, Placebo-Controlled Clinical Trial**

**Danqi Wang^1^, Xiaohua Wang^1^, Jin Han^1^, Chunping You^1^, Zhenmin Liu^1^, and Zhengjun Wu^1, *^**

^1^ State Key Laboratory of Dairy Biotechnology, Shanghai Engineering Research Center of Dairy Biotechnology, Dairy Research Institute, Bright Dairy & Food Co., Ltd., Shanghai 200436, PR China

* **Corresponding author:** Zhengjun Wu, **E-mail:** [wuzhengjun@brightdairy.com](mailto:wuzhengjun@brightdairy.com)

**Table S1. Baseline Characteristics**

|  | ***L. casei* LC2W**  **(n=30)** | **Placebo**  **(n=30)** | **Group Difference**  **p-value** |
| --- | --- | --- | --- |
| Male, n (%) | 18 (60.0) | 12 (40.0) | 0.121 |
| Age, year | 54.2±4.5 | 52.1±3.3 | 0.068 |
| Body weight, kg | 68.4±11.5 | 64.2±7.6 | 0.334 |
| Height, cm | 165.6±7.0 | 161.4±7.4 | 0.111 |
| Body mess index (BMI), kg/m^2^ | 24.9±3.4 | 24.6±1.9 | 0.824 |
| Waist circumference, cm | 94.5±10.9 | 89.1±7.6 | 0.101 |
| Hip circumference, cm | 100.6±5.4 | 98.0±5.6 | 0.076 |
| Waist-to-hip ratio | 0.94±0.08 | 0.91±0.09 | 0.628 |
| Body temperature, °C | 36.1±0.4 | 36.0±0.3 | 0.373 |
| Systolic blood pressure, mmHg | 138.2±12.6 | 137.2±13.4 | 0.703 |
| Diastolic blood pressure, mmHg | 87.0±8.1 | 86.3±7.9 | 0.760 |

Data are presents as mean ± standard deviation or frequency (%). Group difference was evaluated using analysis of variance with adjustment for gender for continuous variables, and chi-square test for categorical variables.

**Table S2. Average Daily Food Intake by 72-hour Dietary Recall**

| **Outcomes** | **Group** | **Baseline** | **3 months** | **6 months** | **3 months vs. Baseline p-value** | **6 months vs. Baseline p-value** | **L. casei LC2W vs. Placebo**  **p-value** | | |
| --- | --- | --- | --- | --- | --- | --- | --- | --- | --- |
|  |  |  |  |  |  |  | **Baseline** | **3 months** | **6 months** |
| Dairy product, ml | L.casei LC2W | 120 (80, 240) | 0 (0, 240) | 120 (0, 280) | 0.308 | 0.944 | 0.857 | 0.410 | 0.194 |
|  | Placebo | 160 (80, 240) | 80 (0, 240) | 240 (160, 240) | 0.471 | 0.442 |  |  |  |
| Cheese, g | L.casei LC2W | 0 (0, 0.3) | 0 (0, 0) | 0 (0, 0.3) | 0.717 | 0.166 | 0.923 | 0.255 | 0.852 |
|  | Placebo | 0 (0, 0.3) | 0 (0, 0) | 0.3 (0, 0.3) | 0.436 | 0.213 |  |  |  |
| Fruit, g | L.casei LC2W | 158 (92, 260) | 200 (100, 252) | 200 (118, 235) | 0.797 | 0.377 | 0.946 | 0.148 | 0.275 |
|  | Placebo | 167 (67, 333) | 133 (0, 233) | 203 (167, 267) | 0.528 | 0.496 |  |  |  |
| Vegetable, g | L.casei LC2W | 392 (267, 467) | 425 (267, 542) | 350 (242, 483) | 0.450 | 1.000 | 0.147 | 0.495 | 0.354 |
|  | Placebo | 367 (300, 533) | 333 (233, 600) | 383 (300, 483) | 0.187 | 0.138 |  |  |  |
| Egg and meat, g | L.casei LC2W | 242 (167, 308) | 233 (158, 325) | 250 (150, 300) | 0.606 | 0.288 | 0.166 | 0.394 | 0.489 |
|  | Placebo | 200 (150, 267) | 217 (133, 283) | 200 (167, 267) | 0.868 | 0.835 |  |  |  |
| Cereal, g | L.casei LC2W | 167 (108, 200) | 233 (117, 250) | 183 (142, 250) | 0.316 | 0.086 | 0.555 | 0.095 | 0.240 |
|  | Placebo | 150 (117, 200) | 150 (100, 217) | 183 (150, 200) | 0.990 | 0.077 |  |  |  |
| Beverage, ml | L.casei LC2W | 520 (340, 620) | 520 (280, 1220) | 500 (320, 640) | 0.174 | 0.815 | 0.134 | 0.269 | 0.601 |
|  | Placebo | 560 (440, 720) | 480 (280, 680) | 440 (320, 640) | 0.139 | 0.052 |  |  |  |
| Seasoning, time | L.casei LC2W | 2.3 (2.0, 3.3) | 2.0 (1.7, 4.5) | 2.1 (1.7, 3.0) | 0.334 | 0.464 | 0.203 | 0.417 | 0.912 |
|  | Placebo | 2.0 (2.0, 3.0) | 2.0 (2.0, 2.5) | 2.3 (1.7, 2.7) | 0.938 | 0.890 |  |  |  |

Q1, the first quartile; Q3, the third quartile.

Data are presented as median (Q1, Q3); group differences at each visit were evaluated using Kruskal Wallis test; the differences of post-intervention versus baseline were evaluated using Wilcoxon signed ranks test.

**Table S3. Physical Activities**

| **Outcomes** | **Group** | **Baseline** | **3 months** | **6 months** | **3 months vs. Baseline p-value** | **6 months vs. Baseline p-value** | **L. casei LC2W vs. Placebo**  **p-value** | | |
| --- | --- | --- | --- | --- | --- | --- | --- | --- | --- |
|  |  |  |  |  |  |  | **Baseline** | **3 months** | **6 months** |
| Vigorous activities, hour/week ^a^ | L.casei LC2W | 0 (0, 0) | 0 (0, 0) | 0 (0, 0) | 0.813 | 0.688 | 0.980 | 0.663 | 0.706 |
|  | Placebo | 0 (0, 0) | 0 (0, 0) | 0 (0, 0) | 1.000 | 0.906 |  |  |  |
| Moderate activities, hour/week ^a^ | L.casei LC2W | 0 (0, 0) | 0 (0, 0) | 0 (0, 0) | 0.688 | 0.625 | 0.419 | 0.950 | 0.578 |
|  | Placebo | 0 (0, 0) | 0 (0, 0) | 0 (0, 0) | 0.750 | 1.000 |  |  |  |
| Walk, hour/week ^a^ | L.casei LC2W | 3.5 (0.0, 11.3) | 3.5 (0.0, 6.4) | 4.7 (0.8, 17.5) | 0.235 | 0.300 | 0.504 | 0.407 | 0.110 |
|  | Placebo | 3.0 (0.0, 7.0) | 4.3 (0.0, 7.0) | 3.5 (0.0, 7.0) | 0.738 | 0.710 |  |  |  |
| Sitting, hour/day | L.casei LC2W | 4.8±1.7 | 5.0±2.3 | 4.2±2.5 | 0.627 | 0.251 | 0.230 | 0.329 | 0.900 |
|  | Placebo | 4.3±1.0 | 4.5±1.2 | 4.2±0.9 | 0.482 | 0.693 |  |  |  |
| Sleep, hour/day | L.casei LC2W | 6.9±1.1 | 7.0±0.9 | 6.8±1.0 | 0.527 | 0.829 | 0.433 | 0.357 | 0.655 |
|  | Placebo | 6.7±0.9 | 6.8±0.7 | 6.7±0.8 | 0.220 | 0.650 |  |  |  |

Q1, the first quartile; Q3, the third quartile.

Unless otherwise stated, data are presented as mean ± standard deviation; group differences at each visit were evaluated using analysis of variance; the differences of post-intervention versus baseline were evaluated using paired t-test.

^a^ Data are presented as median (Q1, Q3); group differences at each visit were evaluated using Kruskal Wallis test; the differences of post-intervention versus baseline were evaluated using Wilcoxon signed ranks test.

**Table S4. Anthropometric Measurements**

| **Outcomes** | **Group** | **Baseline** | **3 months** | **6 months** | **3 months vs. Baseline** | **6 months vs. Baseline** | **L.casei LC2W vs. Placebo** | | |
| --- | --- | --- | --- | --- | --- | --- | --- | --- | --- |
|  |  |  |  |  |  |  | **Baseline** | **3 months** | **6 months** |
| Body weight, kg | L.casei LC2W | 68.7±11.7 | 67.9±11.9 | 67.3±11.5 | -0.8 (-1.7, 0.1) | -1.4 (-2.4, -0.4) | 0.508 | 0.138 | 0.089 |
|  | Placebo | 63.7±7.1 | 63.6±7.0 | 63.8±7.1 | -0.2 (-0.5, 0.2) | 0.04 (-0.5, 0.6) |  |  |  |
| Height, cm | L.casei LC2W | 166.0±6.9 | 165.9±6.9 | 165.9±6.9 | -0.1 (-0.4, 0.1) | -0.1 (-0.3, 0.2) | 0.223 | 0.450 | 0.811 |
|  | Placebo | 160.9±7.4 | 160.8±7.5 | 160.8±7.4 | -0.04 (-0.1, 0.04) | -0.1 (-0.4, 0.2) |  |  |  |
| Body mass index, kg/m^2^ | L.casei LC2W | 24.8±3.4 | 24.6±3.6 | 24.4±3.4 | -0.3 (-0.6, 0.1) | -0.5 (-0.8, -0.1) | 0.983 | 0.171 | 0.044 |
|  | Placebo | 24.6±1.8 | 24.5±1.7 | 24.6±1.7 | -0.1 (-0.2, 0.1) | 0.03 (-0.2, 0.3) |  |  |  |
| Waist circumference, cm | L.casei LC2W | 94.0±11.1 | 93.7±11.2 | 93.6±11.0 | -0.3 (-0.5, 0.04) | -0.3 (-0.7, 0.1) | 0.341 | 0.326 | 0.411 |
|  | Placebo | 88.8±7.3 | 88.7±7.3 | 88.9±7.2 | -0.04 (-0.2, 0.1) | 0.1 (-0.3, 0.5) |  |  |  |
| Hip circumference, cm | L.casei LC2W | 100.2±5.3 | 99.8±5.0 | 99.6±5.8 | -0.4 (-1.2, 0.4) | -0.5 (-1.5, 0.4) | 0.100 | 0.200 | 0.102 |
|  | Placebo | 97.6±5.8 | 98.1±5.6 | 97.8±5.2 | 0.5 (-0.5, 1.5) | 0.2 (-0.6, 1.0) |  |  |  |
| Waist-to-hip ratio | L.casei LC2W | 0.94±0.08 | 0.94±0.08 | 0.94±0.08 | 0.001 (-0.01, 0.01) | 0.002 (-0.01, 0.01) | 0.751 | 0.846 | 0.805 |
|  | Placebo | 0.91±0.09 | 0.91±0.09 | 0.91±0.08 | -0.005 (-0.01, 0.004) | -0.001 (-0.01, 0.01) |  |  |  |

Unless otherwise stated, data are presented as mean ± standard deviation. Differences between groups are presented as mean (95% confidence interval) and evaluated using analysis of variance. Differences between post-intervention and baseline measurements are presented as mean (95% confidence interval) and evaluated by paired t-test.

| **Table S5. Anthropometric Measurements for female and male subgroups** | | | | | | | | | | | | |
| --- | --- | --- | --- | --- | --- | --- | --- | --- | --- | --- | --- | --- |
| **Outcomes** | **Gender** | **Group** | **Baseline** | **3 months** | **6 months** | **3 months vs. Baseline** | **6 months vs. Baseline** | **L.casei LC2W vs. Placebo** | | | **Female vs. Male** | |
|  |  |  |  |  |  |  |  | **Baseline** | **3 months** | **6 months** | **3M-BL** | **6M-BL** |
| Body weight, kg | Female | L.casei LC2W | 61.1±9.17 | 59.5±9.20 | 59.9±8.62 | -1.6 (-3.3, 0.2) | -1.2 (-3.3, 1.0) | 0.412 | 0.387 | 0.402 | 0.141 | 0.379 |
|  |  | Placebo | 60.4±5.94 | 60.3±5.99 | 60.6±6.10 | -0.1 (-0.5, 0.3) | 0.2 (-0.6, 0.9) |  |  |  |  |  |
|  | Male | L.casei LC2W | 72.9±11.0 | 72.5±10.9 | 71.4±11.0 | -0.4 (-1.3, 0.5) | -1.5 (-2.5, -0.5) | 0.252 | 0.260 | 0.370 |  |  |
|  |  | Placebo | 70.3±3.71 | 70.1±3.51 | 70.1±4.13 | -0.3 (-0.9, 0.3) | -0.2 (-1.1, 0.6) |  |  |  |  |  |
| Height, cm | Female | L.casei LC2W | 159.8±5.41 | 159.8±5.41 | 159.9±5.47 | 0.0 (0.0, 0.0) | 0.1 (-0.3, 0.5) | 0.055 | 0.055 | 0.052 | 0.108 | 0.156 |
|  |  | Placebo | 156.4±4.19 | 156.4±4.31 | 156.4±4.25 | -0.1 (-0.2, 0.1) | 0.0 (-0.3, 0.3) |  |  |  |  |  |
|  | Male | L.casei LC2W | 169.4±5.03 | 169.2±5.11 | 169.3±5.21 | -0.2 (-0.6, 0.1) | -0.2 (-0.5, 0.2) | 0.453 | 0.408 | 0.466 |  |  |
|  |  | Placebo | 169.7±3.43 | 169.7±3.43 | 169.4±3.78 | 0.0 (0.0, 0.0) | -0.2 (-0.9, 0.5) |  |  |  |  |  |
| Body mass index, kg/m^2^ | Female | L.casei LC2W | 23.8±2.74 | 23.3±3.08 | 23.4±2.93 | -0.6 (-1.2, 0.6) | -0.4 (-1.2, 0.3) | 0.205 | 0.089 | 0.051 | 0.098 | 0.454 |
|  |  | Placebo | 24.7±1.89 | 24.6±1.86 | 24.7±1.79 | -0.0 (-0.2, 0.2) | 0.1 (-0.3, 0.4) |  |  |  |  |  |
|  | Male | L.casei LC2W | 25.4±3.69 | 25.3±3.67 | 24.9±3.67 | -0.2 (-0.4, 0.3) | -0.5 (-0.8, 0.1) | 0.239 | 0.227 | 0.360 |  |  |
|  |  | Placebo | 24.5±1.60 | 24.4±1.36 | 24.4±1.66 | -0.1 (-0.3, 0.1) | -0.0 (-0.4, 0.3) |  |  |  |  |  |
| Waist circumference, cm | Female | L.casei LC2W | 86.2±6.81 | 86.2±6.53 | 86.2±6.68 | -0.2 (-0.5, 0.1) | 0.0 (-0.6, 0.6) | 0.421 | 0.398 | 0.383 | 0.379 | 0.110 |
|  |  | Placebo | 86.6±6.95 | 86.7±7.00 | 86.8±6.93 | 0.1 (-0.1, 0.2) | 0.2 (-0.3, 0.6) |  |  |  |  |  |
|  | Male | L.casei LC2W | 98.3±10.8 | 98.0±11.0 | 97.8±10.9 | -0.3 (-0.7, 0.1) | -0.5 (-1.0, 0.0) | 0.100 | 0.105 | 0.117 |  |  |
|  |  | Placebo | 93.1±6.21 | 92.9±6.41 | 93.0±5.96 | -0.2 (-0.5, 0.1) | -0.1 (-0.9, 0.7) |  |  |  |  |  |
| Hip circumference, cm | Female | L.casei LC2W | 98.7±3.16 | 98.2±4.05 | 97.3±5.62 | -0.5 (-2.2, 1.2) | -1.4 (-3.7, 0.9) | 0.455 | 0.447 | 0.470 | 0.411 | 0.150 |
|  |  | Placebo | 98.6±5.88 | 98.5±5.99 | 98.1±5.40 | -0.1 (-1.3, 1.0) | -0.6 (-1.4, 0.3) |  |  |  |  |  |
|  | Male | L.casei LC2W | 101±6.17 | 101±5.42 | 101±5.62 | -0.3 (-1.0, 0.5) | -0.1 (-0.6, 0.5) | 0.017 | 0.06 | 0.054 |  |  |
|  |  | Placebo | 95.6±5.39 | 97.2±5.09 | 97.2±5.17 | 1.7 (0.3, 3.1) | 1.7 (0.4, 2.9) |  |  |  |  |  |
| Waist-to-hip ratio | Female | L.casei LC2W | 0.87±0.06 | 0.88±0.06 | 0.89±0.06 | 0.003 (-0.013, 0.019) | 0.014 (-0.012, 0.040) | 0.363 | 0.324 | 0.341 | 0.374 | 0.107 |
|  |  | Placebo | 0.88±0.06 | 0.88±0.06 | 0.89±0.07 | 0.002 (-0.009, 0.012) | 0.007 (-0.002, 0.016) |  |  |  |  |  |
|  | Male | L.casei LC2W | 0.97±0.07 | 0.97±0.07 | 0.97±0.07 | 0.00 (-0.008, 0.007) | -0.004 (-0.011, 0.003) | 0.420 | 0.361 | 0.406 |  |  |
|  |  | Placebo | 0.98±0.10 | 0.96±0.10 | 0.96±0.09 | -0.019 (-0.032, -0.006) | -0.019 (-0.037, -0.001) |  |  |  |  |  |
| Unless otherwise stated, data are presented as mean ± standard deviation. Differences between groups are presented as mean (95% confidence interval) and evaluated using analysis of variance. Differences between post-intervention and baseline measurements are presented as mean (95% confidence interval) and evaluated by paired t-test. | | | | | | | | | | | | |
|  |  |  |  |  |  |  |  |  |  |  |  |  |

**Table S6. Blood Biomarkers**

| **Outcomes** | **Group** | **Baseline** | **3 months** | **6 months** | **3 months vs. Baseline** | **6 months vs. Baseline** | ***L. casei* LC2W vs. Placebo** | | |
| --- | --- | --- | --- | --- | --- | --- | --- | --- | --- |
|  |  |  |  |  |  |  | **Baseline** | **3 months** | **6 months** |
| **Glucose metabolism markers** |  |  |  |  |  |  |  |  |  |
| Fasting glucose, mmol/L | L. casei LC2W | 6.6±0.2 | 6.5±0.3 | 5.8±0.4 | -0.04 (-0.2, 0.01) | -0.7 (-0.9, -0.6) | 0.832 | 0.191 | <0.0001 |
|  | Placebo | 6.6±0.3 | 6.6±0.3 | 6.6±0.3 | 0.04 (-0.01, 0.1) | 0.01 (-0.1, 0.1) |  |  |  |
| Glucose tolerance-0.5 hour, mmol/L | L. casei LC2W | 12.5±0.8 | 12.1±0.9 | 9.4±1.5 | -0.4 (-0.9, 0.04) | -3.1 (-3.8, -2.5) | 0.758 | 0.061 | <0.0001 |
|  | Placebo | 12.6±0.7 | 12.6±0.7 | 12.6±0.8 | -0.04 (-0.2, 0.1) | -0.01 (-0.2, 0.1) |  |  |  |
| Glucose tolerance-1 hour, mmol/L | L. casei LC2W | 11.5±1.0 | 11.2±1.0 | 8.9±1.4 | -0.3 (-0.8, 0.1) | -2.7 (-3.3, -2.1) | 0.705 | 0.106 | <0.0001 |
|  | Placebo | 11.6±0.8 | 11.6±0.9 | 11.6±0.9 | -0.01 (-0.2, 0.2) | -0.02 (-0.2, 0.1) |  |  |  |
| Glucose tolerance-2 hour, mmol/L | L. casei LC2W | 8.3±0.7 | 8.2±0.8 | 7.0±0.6 | -0.2 (-0.3, 0.02) | -1.4 (-1.6, -1.1) | 0.355 | 0.098 | <0.0001 |
|  | Placebo | 8.5±0.6 | 8.5±0.7 | 8.5±0.6 | 0.02 (-0.1, 0.2) | -0.01 (-0.1, 0.1) |  |  |  |
| Insulin ^a^, μU/ml | L. casei LC2W | 20.1 (11.0, 31.5) | 19.3 (11.8, 28.0) | 11.1 (9.0, 22.8) | -1.2(-2.0. -0.2) | -4.7 (-11.6, -2.3) | 0.281 | 0.102 | 0.003 |
|  | Placebo | 22.7 (13.6, 33.0) | 22.4 (16.5, 32.9) | 19.9 (14.3, 28.8) | 0.4 (-0.5, 2.2) | -0.01 (-1.7, 1.7) |  |  |  |
| Glycated hemoglobin ^a^, % | L. casei LC2W | 6.2 (6.1, 6.3) | 6.1 (5.8, 6.2) | 5.6 (5.5, 5.8) | -0.04 (-0.2, 0.1) | -0.5 (-0.8, -0.2) | 0.933 | 0.083 | <0.0001 |
|  | Placebo | 6.2 (5.9, 6.4) | 6.2 (6.0, 6.3) | 6.2 (5.9, 6.3) | 0.1 (-0.03, 0.1) | -0.01 (-0.1, 0.1) |  |  |  |
|  |  |  |  |  |  |  |  |  |  |
| **Lipids** |  |  |  |  |  |  |  |  |  |
| Triglyceride mmol/L | L. casei LC2W | 2.3±1.0 | 1.9±0.7 | 1.8±0.8 | -0.4 (-0.7, -0.2) | -0.5 (-0.8, -0.3) | 0.898 | 0.040 | 0.013 |
|  | Placebo | 2.3±0.9 | 2.3±0.9 | 2.3±0.9 | -0.01 (-0.1, 0.1) | 0.02 (-0.1, 0.1) |  |  |  |
| Total cholesterol, mmol/L | L. casei LC2W | 5.6±1.1 | 5.2±0.8 | 5.0±0.9 | -0.4 (-0.6, -0.3) | -0.5 (-0.8, -0.3) | 0.923 | 0.041 | 0.016 |
|  | Placebo | 5.6±1.0 | 5.7±0.9 | 5.7±1.0 | 0.04 (-0.02, 0.1) | 0.05 (-0.02, 0.1) |  |  |  |
| HDL-cholesterol, mmol/L | L. casei LC2W | 1.2±0.2 | 1.4±0.3 | 1.4±0.3 | 0.2 (0.1, 0.3) | 0.25 (0.2, 0.3) | 0.474 | 0.038 | 0.005 |
|  | Placebo | 1.2±0.2 | 1.2±0.2 | 1.2±0.3 | 0.01 (-0.02, 0.1) | 0.001 (-0.1, 0.1) |  |  |  |
| LDL-cholesterol mmol/L | L. casei LC2W | 3.0±0.5 | 3.0±0.5 | 2.6±0.5 | -0.04 (-0.1, 0.04) | -0.5 (-0.6, -0.3) | 0.754 | 0.778 | 0.0007 |
|  | Placebo | 3.1±0.6 | 3.0±0.6 | 3.1±0.5 | -0.05 (-0.1, 0.01) | -0.01 (-0.1, 0.1) |  |  |  |
|  |  |  |  |  |  |  |  |  |  |
| **Inflammation** **markers** |  |  |  |  |  |  |  |  |  |
| High-sensitivity CRP ^a^, mg/L | L. casei LC2W | 1.3 (0.7, 3.1) | 0.7 (0.5, 1.4) | 0.6 (0.5, 1.2) | -0.3 (-0.7, -0.1) | -0.4 (-1.3, -0.1) | 0.596 | 0.007 | 0.0002 |
|  | Placebo | 1.8 (0.8, 2.9) | 1.5 (0.7, 2.9) | 1.7 (0.9, 3.2) | 0.02 (-0.2, 0.2) | 0.02 (-0.1, 0.3) |  |  |  |
| Interleukin-6, pg/ml | L. casei LC2W | 3.7±0.3 | 3.4±0.449 | 3.1±0.5 | -0.4 (-0.6, -0.2) | -0.6 (-0.9, -0.4) | 0.079 | <0.0001 | <0.0001 |
|  | Placebo | 3.9±0.4 | 3.9±0.5 | 3.9±0.6 | -0.01 (-0.1, 0.1) | 0.02 (-0.1, 0.1) |  |  |  |
| Interleukin-8, pg/ml | L. casei LC2W | 45.8±15.4 | 43.8±12.3 | 42.9±11.9 | -1.9(-4.7, 0.8) | -2.9 (-6.3, 0.5) | 0.916 | 0.489 | 0.232 |
|  | Placebo | 46.2±15.2 | 46.4±15.4 | 47.5±16.2 | 0.2 (-1.6, 2.0) | 1.2 (-1.4, 3.9) |  |  |  |
| Tumour necrosis factor-α, pg/ml | L. casei LC2W | 7.8±0.8 | 7.5±0.7 | 7.5±0.6 | -0.2 (-0.5, 0.02) | -0.3 (-0.5, 0.01) | 0.953 | 0.450 | 0.363 |
|  | Placebo | 7.7±1.4 | 7.8±1.5 | 7.8±1.2 | 0.02 (-0.2, 0.2) | 0.01 (-0.2, 0.2) |  |  |  |
|  |  |  |  |  |  |  |  |  |  |
| **Oxidative stress markers** |  |  |  |  |  |  |  |  |  |
| Malondialdehyde, nmol/ml | L. casei LC2W | 6.6±1.7 | 5.6±1.2 | 5.1±1.0 | -1.1 (-1.5, -0.6) | -1.5(-2.1, -0.9) | 0.856 | 0.029 | 0.0003 |
|  | Placebo | 6.5±1.7 | 6.5±1.5 | 6.5±1.6 | -0.03 (-0.2, 0.2) | -0.1(-0.2, 0.1) |  |  |  |
| Superoxide dismutase, U/ml | L. casei LC2W | 128.3±15.3 | 147.1±22.0 | 150.7±24.6 | 18.9 (13.0, 24.7) | 22.4 (16.4, 28.4) | 0.312 | 0.023 | 0.001 |
|  | Placebo | 132.4±14.9 | 135.4±14.4 | 130.4±15.0 | 2.9 (-1.9, 7.7) | -2.0 (-8.0, 4.0) |  |  |  |

CRP, C-reactive protein; HDL, high-density lipoprotein; LDL, low-density lipoprotein; Q1, the first quartile; Q3, the third quartile.

Unless otherwise stated, data are presented as mean ± standard deviation. Differences between groups are presented as mean (95% confidence interval) and evaluated using analysis of variance. Differences between post-intervention and baseline measurements are presented as mean (95% confidence interval) and evaluated by paired t-test.

^a.^Data are presented as median (Q1, Q3). Differences between groups are presented as median (Q1, Q3) and evaluated using Kruskal Wallis test. Differences between post-intervention and baseline measurements are presented as median (Q1, Q3) and evaluated by Wilcoxon signed rank test.

**Table S7. Blood Biomarkers for female and male subgroups**

| **Outcomes** | **Gender** | **Group** | **Baseline** | **3 months** | **6 months** | **3 months vs. Baseline** | **6 months vs. Baseline** | ***L. casei* LC2W vs. Placebo** | | | **Female vs. Male** | |
| --- | --- | --- | --- | --- | --- | --- | --- | --- | --- | --- | --- | --- |
|  |  |  |  |  |  |  |  | **Baseline** | **3 months** | **6 months** | **3M-BL** | **6M-BL** |
| **Glucose metabolism markers** | | | | | | | | | | |  |  |
| Fasting glucose, mmol/L | Female | L. casei LC2W | 6.47±0.20 | 6.40±0.23 | 5.75±0.39 | -0.07 (-0.11, 0.03) | -0.72 (-0.93, -0.51) | 0.075 | 0.007 | <0.0001 | 0.159 | 0.414 |
|  |  | Placebo | 6.63±0.30 | 6.64±0.27 | 6.65±0.36 | 0.04 (-0.02, 0.09) | 0.02 (-0.06, 0.10) |  |  |  |  |  |
|  | Male | L. casei LC2W | 6.61±0.22 | 6.50±0.26 | 5.86±0.35 | -0.03 (-0.10, 0.04) | -0.75 (-0.91, -0.59) | 0.054 | 0.275 | <0.0001 |  |  |
|  |  | Placebo | 6.47±0.18 | 6.52±0.29 | 6.47±0.29 | 0.04 (-0.04, 0.13) | -0.01 (-0.10, 0.10) |  |  |  |  |  |
| Glucose tolerance-0.5 hour, mmol/L | Female | L. casei LC2W | 12.1±0.89 | 12.1±0.78 | 9.54±1.28 | -0.00 (-0.85, 0.84) | -2.55 (-3.58, -1.53) | 0.097 | 0.114 | <0.0001 | 0.111 | 0.093 |
|  |  | Placebo | 12.5±0.80 | 12.5±0.77 | 12.6±0.80 | -0.06 (-0.26, 0.13) | 0.03 (-0.11, 0.17) |  |  |  |  |  |
|  | Male | L. casei LC2W | 12.8±0.64 | 12.2±1.03 | 9.32±1.72 | -0.63 (-1.08, -0.17) | -3.47 (-4.30, -2.65) | 0.463 | 0.055 | <0.0001 |  |  |
|  |  | Placebo | 12.8±0.62 | 12.8±0.51 | 12.9±0.86 | 0.00 (-0.23, 0.23) | -0.09 (-0.39, 0.21) |  |  |  |  |  |
| Glucose tolerance-1 hour, mmol/L | Female | L. casei LC2W | 10.9±0.88 | 11.0±1.03 | 9.14±1.02 | 0.06 (-0.72, 0.85) | -1.80 (-2.54, -1.06) | 0.083 | 0.179 | <0.0001 | 0.096 | 0.007 |
|  |  | Placebo | 11.5±0.90 | 11.4±1.02 | 11.5±0.92 | -0.07 (-0.26, 0.13) | 0.08 (-0.08, 0.23) |  |  |  |  |  |
|  | Male | L. casei LC2W | 11.8±0.95 | 11.3±0.96 | 8.69±1.53 | -0.56 (-0.99, 0.14) | -3.16 (-3.83, -2.49) | 0.373 | 0.018 | <0.0001 |  |  |
|  |  | Placebo | 12.0±0.57 | 12.1±0.63 | 11.7±0.87 | 0.11 (-0.26, 0.47) | -0.21 (-0.53, 0.11) |  |  |  |  |  |
| Glucose tolerance-2 hour, mmol/L | Female | L. casei LC2W | 8.14±0.62 | 8.07±0.68 | 6.90±0.76 | -0.08 (-0.41, 0.26) | -1.26 (-1.81, -0.70) | 0.078 | 0.041 | <0.0001 | 0.251 | 0.315 |
|  |  | Placebo | 8.48±0.57 | 8.48±0.52 | 8.54±0.55 | -0.03 (-0.14, 0.13) | 0.54 (-0.07, 0.18) |  |  |  |  |  |
|  | Male | L. casei LC2W | 8.44±0.74 | 8.23±0.90 | 7.03±0.53 | -0.21 (-0.41, -0.01) | -1.41 (-1.63, -1.18) | 0.37 | 0.164 | <0.0001 |  |  |
|  |  | Placebo | 8.54±0.79 | 8.62±1.10 | 8.40±0.77 | 0.08 (-0.20, 0.36) | -0.014 (-0.32, 0.04) |  |  |  |  |  |
| Insulin ^a^, μU/ml | Female | L. casei LC2W | 13.1 (11.0, 19.8) | 12.8 (11.3, 19.5) | 9.39 (8.39, 16.3) | -0.47(-2.15, 1.21) | -4.58 (-6.56, -2.60) | 0.013 | 0.002 | 0.001 | 0.285 | 0.027 |
|  |  | Placebo | 25.1 (14.5, 33.7) | 25.4 (19.3, 33.5) | 24.1 (15.3, 32.9) | 0.84 (-0.59, 2.29) | -0.52 (-3.10, 2.07) |  |  |  |  |  |
|  | Male | L. casei LC2W | 26.1 (11.9, 33.8) | 21.8 (13.6, 32.4) | 14.1 (9.62, 23.7) | -1.18(-2.94, 0.57) | -8.61 (-12.0, -5.25) | 0.143 | 0.262 | 0.406 |  |  |
|  |  | Placebo | 17.6 (13.9, 22.7) | 19.1 (15.1, 22.2) | 15.2 (13.6, 19.4) | 0.86 (-0.14, 1.86) | -3.16 (-8.34, 2.02) |  |  |  |  |  |
| Glycated hemoglobin ^a^, % | Female | L. casei LC2W | 6.27 (6.08, 6.30) | 6.06 (5.88, 6.11) | 5.60 (5.52, 5.68) | -0.15 (-0.28, -0.03) | -0.57 (-0.77, -0.37) | 0.214 | 0.057 | <0.0001 | 0.044 | 0.197 |
|  |  | Placebo | 6.04 (5.95, 6.30) | 6.18 (5.96, 6.27) | 6.16 (5.91, 6.31) | 0.04 (-0.02, 0.09) | 0.02 (-0.02, 0.06) |  |  |  |  |  |
|  | Male | L. casei LC2W | 6.14 (6.10, 6.20) | 6.09 (5.89, 6.28) | 5.75 (5.45, 5.88) | -0.02 (-0.09, 0.04) | -0.46 (-0.61, -0.31) | 0.229 | 0.097 | <0.0001 |  |  |
|  |  | Placebo | 6.25 (5.99, 6.34) | 6.23 (6.18, 6.34) | 6.23 (6.16, 6.28) | 0.03 (-0.05, 0.11) | -0.01 (-0.10, 0.08) |  |  |  |  |  |
| **Lipids** |  |  |  |  |  |  |  |  |  |  |  |  |
| Triglyceride mmol/L | Female | L. casei LC2W | 2.26±1.15 | 1.67±0.80 | 1.58±0.85 | -0.58 (-1.12, -0.05) | -0.68 (-1.25, -0.10) | 0.499 | 0.039 | 0.022 | 0.226 | 0.257 |
|  |  | Placebo | 2.26±0.87 | 2.28±0.86 | 2.34±0.93 | 0.02 (-0.05, 0.10) | 0.08 (-0.05, 0.21) |  |  |  |  |  |
|  | Male | L. casei LC2W | 2.30±0.93 | 1.95±0.69 | 1.84±0.71 | -0.36 (-0.54, -0.18) | -0.47 (-0.68, -0.25) | 0.363 | 0.103 | 0.07 |  |  |
|  |  | Placebo | 2.45±1.12 | 2.38±1.03 | 2.35±1.04 | -0.07 (-0.27, 0.13) | -0.09 (-0.23, 0.04) |  |  |  |  |  |
| Total cholesterol, mmol/L | Female | L. casei LC2W | 5.67±1.20 | 5.11±0.90 | 4.75±0.75 | -0.56 (-0.83, -0.29) | -0.92 (-1.37, -0.46) | 0.469 | 0.059 | 0.007 | 0.119 | 0.019 |
|  |  | Placebo | 5.71±1.12 | 5.75±1.06 | 5.79±1.12 | 0.05 (-0.03, 0.12) | 0.09 (0.01, 0.16) |  |  |  |  |  |
|  | Male | L. casei LC2W | 5.53±1.09 | 5.17±0.82 | 5.20±0.89 | -0.36 (-0.54, -0.16) | -0.35 (-0.51, -0.16) | 0.385 | 0.192 | 0.277 |  |  |
|  |  | Placebo | 5.41±0.66 | 5.45±0.67 | 5.40±0.69 | 0.03 (-0.04, 0.10) | -0.02 (-0.16, 0.12) |  |  |  |  |  |
| HDL-cholesterol, mmol/L | Female | L. casei LC2W | 1.15±0.21 | 1.47±0.33 | 1.51±0.31 | 0.33 (0.16, 0.49) | 0.36 (0.22, 0.50) | 0.193 | 0.024 | 0.007 | 0.028 | 0.031 |
|  |  | Placebo | 1.23±0.25 | 1.24±0.26 | 1.21±0.28 | 0.00 (-0.03, 0.04) | -0.02 (-0.08, 0.03) |  |  |  |  |  |
|  | Male | L. casei LC2W | 1.15±0.18 | 1.28±0.20 | 1.34±0.22 | 0.13 (0.07, 0.19) | 0.19 (0.10, 0.28) | 0.296 | 0.05 | 0.029 |  |  |
|  |  | Placebo | 1.11±0.18 | 1.14±0.20 | 1.16±0.23 | 0.03 (-0.04, 0.10) | 0.05 (-0.02, 0.12) |  |  |  |  |  |
| LDL-cholesterol mmol/L | Female | L. casei LC2W | 3.12±0.39 | 3.12±0.37 | 2.72±0.31 | -0.00 (-0.12, 0.12) | -0.40 (-0.58, -0.22) | 0.475 | 0.406 | 0.017 | 0.232 | 0.245 |
|  |  | Placebo | 3.13±0.61 | 3.07±0.59 | 3.10±0.49 | -0.07 (-0.15, 0.01) | -0.03 (-0.14, 0.07) |  |  |  |  |  |
|  | Male | L. casei LC2W | 2.96±0.53 | 2.90±0.52 | 2.48±0.57 | -0.06 (-0.17, 0.04) | -0.49 (-0.65, -0.33) | 0.439 | 0.467 | 0.023 |  |  |
|  |  | Placebo | 2.93±0.58 | 2.92±0.55 | 2.96±0.56 | -0.01 (-0.06, 0.04) | 0.03 (-0.05, 0.11) |  |  |  |  |  |
| **Inflammation** **markers** | | | | | | | | | | |  |  |
| High-sensitivity CRP ^a^, mg/L | Female | L. casei LC2W | 1.31 (0.80, 2.51) | 0.65 (0.50, 1.22) | 0.50 (0.50, 1.02) | -0.85 (-1.56, -0.14) | -0.91 (-1.50, -0.32) | 0.314 | 0.014 | 0.003 | 0.276 | 0.234 |
|  |  | Placebo | 1.78 (1.00, 2.62) | 1.54 (1.06, 2.57) | 1.72 (1.11, 2.81) | -0.00 (-0.15, 0.14) | 0.12 (-0.02, 0.27) |  |  |  |  |  |
|  | Male | L. casei LC2W | 1.29 (0.63, 2.99) | 0.96 (0.50, 1.78) | 0.61 (0.50, 1.44) | -1.45 (-3.23, 0.36) | -1.64 (-3.47, 0.20) | 0.233 | 0.017 | 0.01 |  |  |
|  |  | Placebo | 1.88 (0.85, 3.85) | 2.19 (0.83, 3.42) | 2.09 (0.91, 1.18) | -0.74 (-2.90, 1.42) | -1.06 (-3.20, 1.08) |  |  |  |  |  |
| Interleukin-6, pg/ml | Female | L. casei LC2W | 3.55±0.11 | 3.41±0.17 | 3.25±0.24 | -0.14 (-0.21, -0.07) | -0.30 (-0.44, -0.16) | 0.004 | 0.0001 | 0.001 | 0.014 | 0.005 |
|  |  | Placebo | 3.99±0.48 | 3.97±0.59 | 4.05±0.69 | -0.02 (-0.21, 0.03) | 0.06 (-0.08, 0.21) |  |  |  |  |  |
|  | Male | L. casei LC2W | 3.81±0.31 | 3.31±0.48 | 2.98±0.53 | -0.50 (-0.78, -0.21) | -0.82 (-1.15, -0.49) | 0.155 | 0.018 | 0.001 |  |  |
|  |  | Placebo | 3.69±0.21 | 3.71±0.36 | 3.62±0.18 | 0.03 (-0.09, 0.14) | -0.06 (-0.14, 0.01) |  |  |  |  |  |
| Interleukin-8, pg/ml | Female | L. casei LC2W | 42.9±15.2 | 43.8±12.4 | 42.4±12.6 | 0.89 (-2.01, 3.78) | -0.52 (-3.47, 2.43) | 0.225 | 0.286 | 0.095 | 0.038 | 0.100 |
|  |  | Placebo | 47.5±15.2 | 47.0±15.3 | 49.9±15.0 | -0.46 (-2.26, 1.34) | 2.41 (-1.08, 5.91) |  |  |  |  |  |
|  | Male | L. casei LC2W | 47.4±15.8 | 43.9±12.6 | 43.1±11.8 | -3.52(-7.18, 0.14) | -4.27 (-8.99, 0.45) | 0.286 | 0.403 | 0.464 |  |  |
|  |  | Placebo | 43.7±15.9 | 45.3±16.4 | 42.6±18.2 | 1.58 (-2.17, 5.34) | -1.10 (-3.54, 1.34) |  |  |  |  |  |
| Tumour necrosis factor-α, pg/ml | Female | L. casei LC2W | 7.69±0.87 | 7.19±0.79 | 7.21±0.74 | -0.50 (-1.01, 0.02) | -0.48 (-1.02, 0.07) | 0.401 | 0.174 | 0.123 | 0.087 | 0.128 |
|  |  | Placebo | 7.83±1.60 | 7.75±1.73 | 7.80±1.45 | -0.08 (-0.27, 0.10) | -0.03 (-0.21, 0.15) |  |  |  |  |  |
|  | Male | L. casei LC2W | 7.80±0.77 | 7.71±0.51 | 7.68±0.50 | -0.08 (-0.31, 0.15) | -0.12 (-0.34, 0.10) | 0.247 | 0.394 | 0.435 |  |  |
|  |  | Placebo | 7.56±0.92 | 7.78±0.71 | 7.65±0.43 | 0.21 (-0.10, 0.53) | 0.08 (-0.32, 0.49) |  |  |  |  |  |
| **Oxidative stress markers** | | | | | | | | | | |  |  |
| Malondialdehyde, nmol/ml | Female | L. casei LC2W | 6.74±1.44 | 5.45±10.87 | 5.17±0.73 | -1.30 (-1.85, -0.75) | -1.57 (-2.67, -0.48) | 0.463 | 0.014 | 0.007 | 0.185 | 0.410 |
|  |  | Placebo | 6.80±1.75 | 6.73±1.60 | 6.65±1.68 | -0.07 (-0.27, 0.13) | -0.16 (-0.37, 0.06) |  |  |  |  |  |
|  | Male | L. casei LC2W | 6.52±1.79 | 5.60±1.37 | 5.10±1.07 | -0.92 (-1.52, -0.31) | -1.42(-2.09, -0.75) | 0.208 | 0.251 | 0.024 |  |  |
|  |  | Placebo | 5.94±1.54 | 5.97±1.28 | 6.11±1.42 | 0.04 (-0.38, 0.45) | 0.18 (-0.11, 0.47) |  |  |  |  |  |
| Superoxide dismutase, U/ml | Female | L. casei LC2W | 123±9.85 | 141±22.1 | 148±24.8 | 17.9 (5.89, 29.9) | 24.5 (14.1, 34.9) | 0.022 | 0.373 | 0.007 | 0.415 | 0.310 |
|  |  | Placebo | 134±14.6 | 139±12.4 | 129±13.6 | 4.83 (-0.09, 9.75) | -5.39 (-12.4, 1.64) |  |  |  |  |  |
|  | Male | L. casei LC2W | 131±17.2 | 151±21.7 | 153±25.0 | 19.4 (13.4, 25.3) | 21.3 (14.4, 28.2) | 0.399 | 0.006 | 0.031 |  |  |
|  |  | Placebo | 129±16.0 | 129±16.4 | 134±17.8 | -0.89 (-10.4, 8.60) | 4.78 (-4.12, 13.7) |  |  |  |  |  |

CRP, C-reactive protein; HDL, high-density lipoprotein; LDL, low-density lipoprotein; Q1, the first quartile; Q3, the third quartile.

Unless otherwise stated, data are presented as mean ± standard deviation. Differences between groups are presented as mean (95% confidence interval) and evaluated using analysis of variance. Differences between post-intervention and baseline measurements are presented as mean (95% confidence interval) and evaluated by paired t-test.

^a.^Data are presented as median (Q1, Q3). Differences between groups are presented as median (Q1, Q3) and evaluated using Kruskal Wallis test. Differences between post-intervention and baseline measurements are presented as median (Q1, Q3) and evaluated by Wilcoxon signed rank test.

| **Table S8. Adverse events during the study** | | | |
| --- | --- | --- | --- |
| Adverse events (%) | *L. casei* LC2W | Placebo | *P* |
|  | 12(40.0) | 16(53.33) | 0.438 |
| Otitis media | 0(0.0) | 2(6.7) |  |
| Conjunctivitis | 1(3.3) | 2(6.7) |  |
| Rhinitis | 1(3.3) | 1(3.3) |  |
| Diarrhea | 0(0.0) | 3(10.0) |  |
| Fracture | 0(0.0) | 1(3.3) |  |
| Trauma | 2(6.7) | 0(0.0) |  |
| Cold | 0(0.0) | 2(6.7) |  |
| Rash | 4(13.3) | 2(6.7) |  |
| Beriberi | 3(10.0) | 1(3.3) |  |
| Other | 1(3.3) | 2(6.7) |  |

| **Table S9 Correlation analysis between the content of short-chain fatty acids and blood biomarkers at baseline and 3 months of**  ***L. casei* LC2W intervention** | | | | | | | | |
| --- | --- | --- | --- | --- | --- | --- | --- | --- |
| Spearman Correlation analysis | Acetic Acid | | Propionic Acid | | Butyric Acid | | SCFAs | |
|  | R | *P* | R | *P* | R | *P* | R | *P* |
| **Glucose metabolism markers** |  |  |  |  |  |  |  |  |
| Fasting glucose, mmol/L | -0.065 | 0.503 | 0.011 | 0.909 | -0.151 | 0.114 | -0.116 | 0.228 |
| Glucose tolerance-0.5 hour, mmol/L | -0.116 | 0.229 | 0.002 | 0.986 | -0.139 | 0.147 | -0.086 | 0.370 |
| Glucose tolerance-1 hour, mmol/L | -0.096 | 0.317 | 0.086 | 0.372 | -0.087 | 0.369 | -0.056 | 0.563 |
| Glucose tolerance-2 hour, mmol/L | 0.163 | 0.089 | -0.014 | 0.884 | **-0.290** | **0.002** | -0.001 | 0.994 |
| Insulin, μU/ml | -0.095 | 0.321 | 0.071 | 0.460 | -0.052 | 0.591 | -0.087 | 0.368 |
| Glycated hemoglobin, % | 0.062 | 0.520 | 0.025 | 0.793 | 0.051 | 0.593 | 0.082 | 0.394 |
| **Lipids metabolism markers** |  |  |  |  |  |  |  |  |
| Triglyceride mmol/L | -0.017 | 0.858 | -0.060 | 0.534 | 0.095 | 0.324 | -0.031 | 0.744 |
| Total cholesterol, mmol/L | -0.011 | 0.913 | 0.003 | 0.972 | -0.140 | 0.146 | -0.059 | 0.542 |
| HDL-cholesterol, mmol/L | 0.162 | 0.091 | **0.213** | **0.025** | 0.083 | 0.386 | **0.208** | **0.029** |
| LDL-cholesterol mmol/L | -0.078 | 0.416 | **-0.189** | **0.049** | **-0.219** | **0.022** | -0.150 | 0.119 |
| **Inflammation** **markers** |  |  |  |  |  |  |  |  |
| High-sensitivity CRP, mg/L | -0.074 | 0.440 | -0.104 | 0.280 | **-0.188** | **0.049** | -0.134 | 0.162 |
| Interleukin-6, pg/ml | -0.161 | 0.094 | -0.015 | 0.878 | -0.080 | 0.405 | -0.145 | 0.129 |
| Interleukin-8, pg/ml | -0.020 | 0.836 | -0.146 | 0.129 | -0.073 | 0.445 | -0.052 | 0.587 |
| Tumour necrosis factor-α, pg/ml | 0.031 | 0.745 | -0.072 | 0.458 | 0.101 | 0.294 | 0.000 | 0.998 |
| **Oxidative stress markers** |  |  |  |  |  |  |  |  |
| Malondialdehyde, nmol/ml | -0.153 | 0.110 | -0.134 | 0.163 | -0.087 | 0.365 | -0.168 | 0.079 |
| Superoxide dismutase, U/ml | 0.157 | 0.103 | **0.248** | **0.009** | 0.133 | 0.167 | **0.228** | **0.017** |

Boldfaced figure indicates *P* value < 0.05.

| **Table S10 Correlation analysis between the content of short-chain fatty acids and major discriminatory OTUs at baseline and 3 months of *L. casei* LC2W intervention** | | | | | | | | | |
| --- | --- | --- | --- | --- | --- | --- | --- | --- | --- |
| Spearman Correlation analysis | Acetic Acid | | Propionic Acid | | Butyric Acid | | SCFAs | |  |
|  | R | *P* | R | *P* | R | *P* | R | *P* |  |
| Otu3 | 0.035 | 0.716 | 0.086 | 0.374 | 0.185 | 0.053 | 0.084 | 0.383 |  |
| Otu5 | -0.071 | 0.461 | -0.024 | 0.803 | -0.010 | 0.918 | -0.086 | 0.373 |  |
| Otu8 | 0.040 | 0.681 | 0.043 | 0.655 | 0.037 | 0.698 | 0.026 | 0.790 |  |
| Otu10 | -0.084 | 0.381 | -0.031 | 0.751 | 0.104 | 0.278 | -0.008 | 0.934 |  |
| Otu12 | 0.062 | 0.519 | -0.119 | 0.217 | 0.127 | 0.187 | 0.061 | 0.524 |  |
| Otu14 | 0.132 | 0.168 | 0.159 | 0.097 | **0.239** | **0.012** | **0.203** | **0.033** |  |
| Otu17 | -0.004 | 0.967 | 0.057 | 0.554 | 0.111 | 0.249 | 0.080 | 0.404 |  |
| Otu65 | -0.107 | 0.264 | -0.045 | 0.644 | -0.012 | 0.905 | -0.053 | 0.582 |  |
| Otu67 | 0.026 | 0.786 | 0.160 | 0.096 | -0.080 | 0.404 | 0.076 | 0.427 |  |
| Otu73 | 0.100 | 0.477 | -0.195 | 0.161 | **0.274** | **0.047** | 0.109 | 0.436 |  |
| Otu16 | -0.099 | 0.303 | 0.066 | 0.496 | -0.059 | 0.538 | -0.038 | 0.692 |  |
| Otu26 | 0.019 | 0.845 | -0.066 | 0.492 | 0.041 | 0.672 | -0.014 | 0.884 |  |
| Otu28 | 0.108 | 0.263 | 0.183 | 0.056 | **0.188** | **0.049** | 0.154 | 0.108 |  |
| Otu22 | 0.072 | 0.606 | **-0.318** | **0.020** | 0.054 | 0.701 | -0.089 | 0.525 |  |
| Otu24 | -0.057 | 0.557 | 0.095 | 0.322 | -0.011 | 0.905 | 0.024 | 0.802 |  |
| Otu30 | 0.164 | 0.086 | 0.097 | 0.313 | 0.097 | 0.311 | 0.165 | 0.084 |  |
| Otu32 | -0.066 | 0.492 | -0.162 | 0.091 | -0.172 | 0.072 | -0.134 | 0.162 |  |
| Otu51 | **-0.195** | **0.042** | -0.040 | 0.676 | -0.006 | 0.948 | -0.144 | 0.133 |  |
| Otu54 | -0.006 | 0.953 | -0.085 | 0.378 | 0.027 | 0.780 | -0.029 | 0.760 |  |
| Otu56 | -0.045 | 0.641 | 0.100 | 0.298 | -0.050 | 0.606 | -0.036 | 0.712 |  |
| Otu60 | -0.069 | 0.476 | 0.038 | 0.692 | -0.058 | 0.545 | -0.068 | 0.482 |  |
| Otu62 | -0.115 | 0.232 | **-0.265** | **0.005** | 0.030 | 0.756 | -0.179 | 0.062 |  |
| Otu27 | 0.091 | 0.343 | **0.188** | **0.050** | 0.011 | 0.911 | 0.124 | 0.196 |  |
| Otu40 | 0.050 | 0.604 | 0.136 | 0.158 | -0.019 | 0.841 | 0.140 | 0.145 |  |
| Otu44 | -0.130 | 0.177 | 0.014 | 0.888 | -0.016 | 0.869 | -0.111 | 0.248 |  |
| Otu52 | -0.100 | 0.300 | 0.039 | 0.683 | -0.086 | 0.372 | -0.087 | 0.367 |  |
| Otu34 | 0.104 | 0.278 | 0.172 | 0.072 | -0.015 | 0.873 | 0.111 | 0.250 |  |
| Otu29 | -0.065 | 0.501 | -0.117 | 0.224 | 0.063 | 0.516 | -0.049 | 0.610 |  |
| Otu33 | 0.025 | 0.797 | 0.108 | 0.263 | -0.025 | 0.792 | 0.039 | 0.686 |  |
| Otu39 | 0.093 | 0.335 | 0.138 | 0.152 | -0.023 | 0.808 | 0.097 | 0.313 |  |
| Otu43 | -0.035 | 0.720 | -0.096 | 0.319 | -0.003 | 0.979 | -0.054 | 0.575 |  |
| Otu48 | 0.122 | 0.204 | 0.080 | 0.404 | -0.045 | 0.639 | 0.080 | 0.404 |  |
| Otu63 | -0.012 | 0.904 | 0.054 | 0.577 | 0.097 | 0.313 | 0.027 | 0.782 |  |
| Otu88 | 0.083 | 0.391 | 0.106 | 0.271 | 0.038 | 0.691 | 0.066 | 0.495 |  |
| Otu145 | -0.145 | 0.131 | -0.062 | 0.519 | -0.041 | 0.668 | -0.143 | 0.137 |  |
| Otu148 | 0.107 | 0.268 | 0.075 | 0.439 | -0.069 | 0.475 | 0.070 | 0.465 |  |
| Otu151 | -0.054 | 0.578 | 0.038 | 0.695 | -0.059 | 0.543 | -0.066 | 0.491 |  |
| Otu334 | **-0.212** | **0.026** | -0.054 | 0.578 | -0.100 | 0.299 | **-0.197** | **0.040** |  |
| Otu517 | 0.088 | 0.363 | 0.159 | 0.097 | -0.009 | 0.927 | 0.121 | 0.208 |  |
| Otu525 | 0.047 | 0.625 | 0.057 | 0.552 | 0.134 | 0.163 | 0.091 | 0.343 |  |
| Otu80 | -0.095 | 0.322 | -0.016 | 0.870 | 0.054 | 0.575 | -0.063 | 0.511 |  |
| Otu117 | 0.032 | 0.737 | -0.018 | 0.853 | 0.039 | 0.684 | 0.028 | 0.768 |  |

Boldfaced figure indicates *P* value < 0.05.

| **Table S11 Correlation analysis between the content of short-chain fatty acids and major discriminatory pathways at baseline and 3 months of *L. casei* LC2W intervention** | | | | | | | | |
| --- | --- | --- | --- | --- | --- | --- | --- | --- |
| **Spearman Correlation analysis** | **Acetic Acid** | | **Propionic Acid** | | **Butyric Acid** | | **SCFAs** | |
|  | R | *P* | R | *P* | R | *P* | R | *P* |
| PWY-5347 | -0.032 | 0.739 | -0.027 | 0.780 | 0.158 | 0.099 | 0.013 | 0.896 |
| HOMOSER-METSYN-PWY | -0.022 | 0.824 | -0.025 | 0.794 | 0.177 | 0.065 | 0.031 | 0.749 |
| MET-SAM-PWY | -0.031 | 0.749 | -0.032 | 0.741 | 0.161 | 0.093 | 0.022 | 0.822 |
| FAO-PWY | 0.048 | 0.618 | -0.025 | 0.796 | 0.095 | 0.321 | 0.067 | 0.488 |
| PWY-6629 | 0.048 | 0.619 | -0.053 | 0.585 | 0.132 | 0.168 | 0.076 | 0.428 |
| LACTOSECAT-PWY | -0.065 | 0.500 | 0.066 | 0.493 | -0.005 | 0.961 | 0.026 | 0.789 |
| FERMENTATION-PWY | -0.126 | 0.191 | -0.130 | 0.177 | **0.165** | **0.037** | -0.089 | 0.354 |
| GLUCOSE1PMETAB-PWY | -0.017 | 0.864 | -0.043 | 0.653 | 0.166 | 0.083 | 0.057 | 0.553 |
| ANAEROFRUCAT-PWY | 0.068 | 0.480 | 0.130 | 0.177 | 0.112 | 0.245 | 0.123 | 0.200 |
| TEICHOICACID-PWY | -0.019 | 0.845 | -0.102 | 0.290 | -0.165 | 0.085 | -0.095 | 0.326 |
| PWY-7210 | -0.010 | 0.918 | -0.155 | 0.107 | **-0.260** | **0.006** | -0.132 | 0.170 |
| PWY-5659 | -0.106 | 0.271 | -0.125 | 0.195 | -0.009 | 0.927 | -0.146 | 0.129 |
| ARGORNPROST-PWY | -0.035 | 0.714 | 0.039 | 0.688 | 0.040 | 0.679 | 0.042 | 0.666 |
| PWY-7242 | -0.035 | 0.715 | -0.094 | 0.326 | -0.066 | 0.495 | -0.079 | 0.411 |
| PWY-6263 | -0.080 | 0.406 | 0.029 | 0.760 | -0.042 | 0.661 | -0.029 | 0.767 |
| PWY-7237 | -0.169 | 0.078 | -0.020 | 0.837 | -0.007 | 0.938 | -0.125 | 0.192 |
| PWY-5913 | **-0.203** | **0.033** | -0.146 | 0.127 | 0.129 | 0.178 | -0.138 | 0.150 |
| SO4ASSIM-PWY | 0.031 | 0.745 | -0.073 | 0.446 | 0.158 | 0.098 | 0.041 | 0.671 |
| SULFATE-CYS-PWY | 0.029 | 0.764 | -0.076 | 0.428 | 0.156 | 0.103 | 0.037 | 0.700 |
| PWY-6572 | -0.153 | 0.112 | -0.035 | 0.716 | 0.035 | 0.714 | -0.128 | 0.184 |
| PWY-7371 | -0.098 | 0.309 | 0.028 | 0.773 | -0.055 | 0.569 | -0.037 | 0.699 |
| PWY-5676 | 0.024 | 0.803 | 0.108 | 0.263 | -0.109 | 0.258 | 0.024 | 0.807 |
| PWY-6353 | -0.133 | 0.167 | **-0.215** | **0.024** | -0.060 | 0.536 | **-0.206** | **0.031** |
| PWY-6588 | -0.018 | 0.853 | 0.066 | 0.494 | -0.053 | 0.581 | -0.005 | 0.961 |
| SALVADEHYPOX-PWY | **-0.195** | **0.041** | **-0.269** | **0.005** | -0.083 | 0.390 | **-0.267** | **0.005** |

Boldfaced figure indicates *P* value < 0.05.


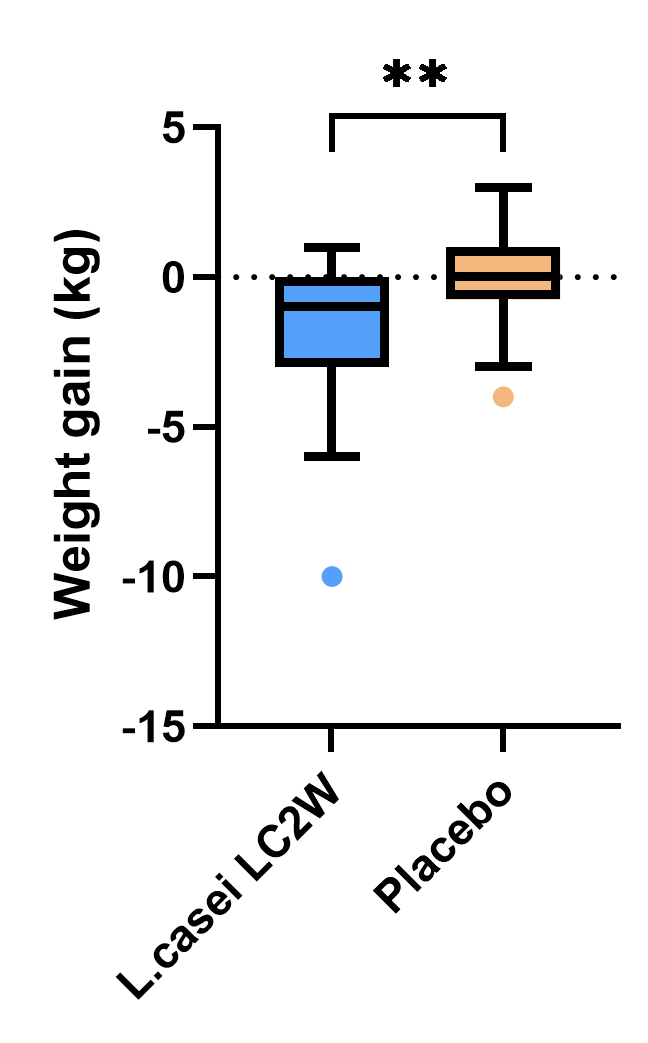


**Fig S1. Effects of 6 months *L. casei* LC2W supplementation on weight gain for all subjects.** ‘*’ donates a significant difference compared to placebo, ** *P* < 0.01.


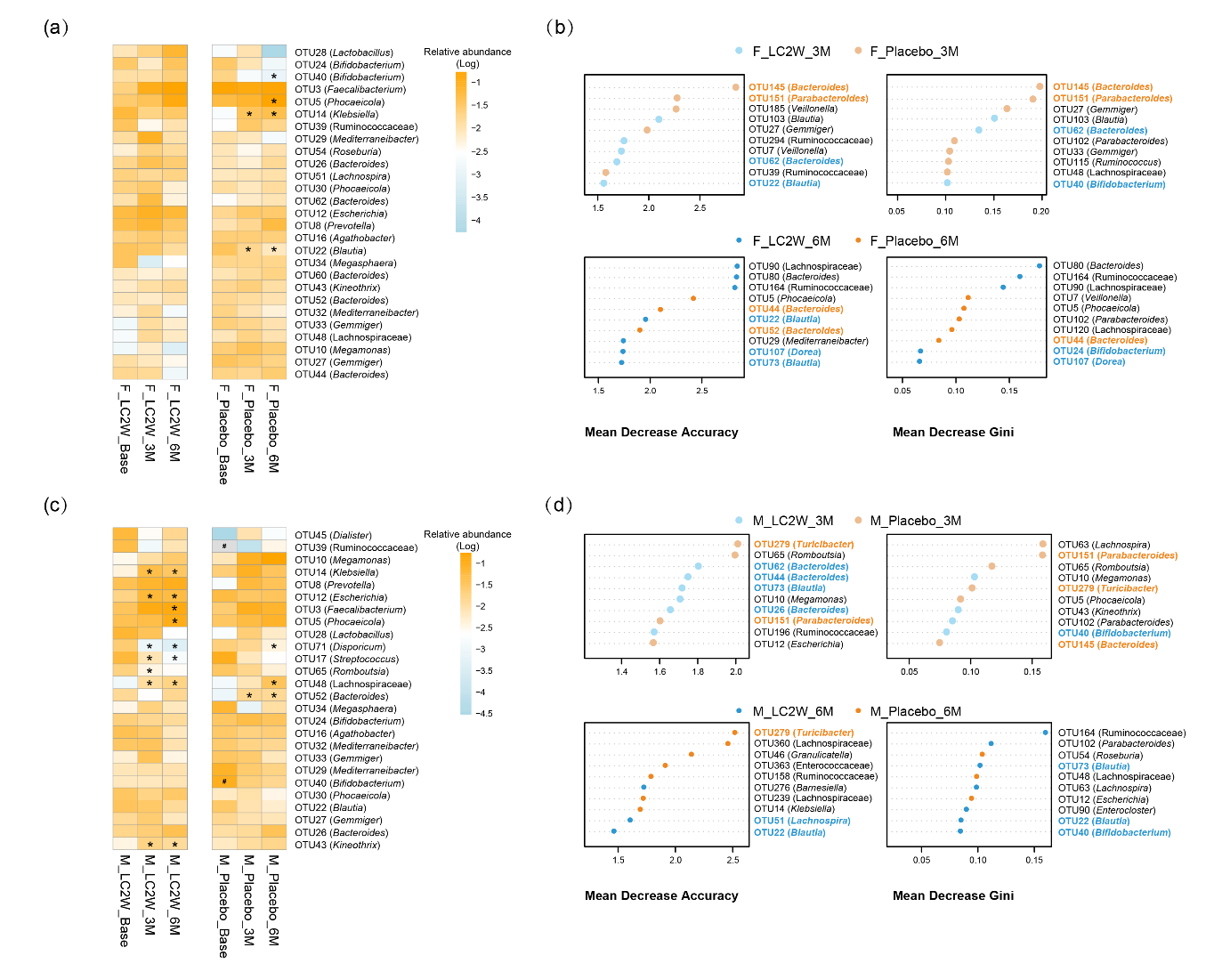


**Fig S2. Bacterial heatmap of major OTUs (relative abundance >1%) of female (a) and male (c) subjects according to 6 groups.** ‘*’ indicates *P* < 0.05 versus baseline in the Kruskal-Wallis test adjusted by the Bonferroni method. ‘#’ donates *P* < 0.05 versus placebo group by Wilcoxon test. **Top 10 OTUs with the highest mean decrease accuracy and Gini scores by Random Forest classification model of female (b) and male (d) subjects for 3 months and 6 months datasets.** The color of the dots represents the enrichment of OTUs in each group by the median.
